# ALS-linked SOD1 in glial cells enhances ß-N-Methylamino L-Alanine (BMAA)-induced toxicity in *Drosophila*

**DOI:** 10.12688/f1000research.1-47.v1

**Published:** 2012-11-09

**Authors:** Rafique Islam, Emily L Kumimoto, Hong Bao, Bing Zhang

**Affiliations:** 1 Department of Biology, University of Oklahoma, Norman, OK, 73019, USA

## Abstract

Environmental factors have been implicated in the etiology of a number of neurodegenerative diseases, including amyotrophic lateral sclerosis (ALS). However, the role of environmental agents in ALS remains poorly understood. To this end, we used transgenic fruit flies (Drosophila melanogaster) to explore the interaction between mutant superoxide dismutase 1 (SOD1) and chemicals such as ß-N-methylamino L-alanine (BMAA), the herbicide agent paraquat, and superoxide species. We expressed ALS-linked human SOD1 (hSOD1A4V, and hSOD1G85R), hSOD1wt as well as the Drosophila native SOD1 (dSOD1) in motoneurons (MNs) or in glial cells alone and simultaneously in both types of cells. We then examined the effect of BMAA (3 mM), paraquat (20 mM), and hydrogen peroxide (H2O2, 1%) on the lifespan of SOD1-expressing flies. Our data show that glial expression of mutant and wild type hSOD1s reduces the ability of flies to climb. Further, we show that while all three chemicals significantly shorten the lifespan of flies, mutant SOD1 does not have a significant additional effect on the lifespan of flies fed on paraquat, but further shortens the lifespan of flies fed on H2O2. Finally, we show that BMAA shows a dramatic cell-type specific effect with mutant SOD1. Flies with expression of mutant hSOD1 in MNs survived longer on BMAA compared to control flies. In contrast, BMAA significantly shortened the lifespan of flies expressing mutant hSOD1 in glia. Consistent with a neuronal protection role, flies expressing these mutant hSOD1s in both MNs and glia also lived longer. Hence, our studies reveal a synergistic effect of mutant SOD1 with H2O2 and novel roles for mutant hSOD1s in neurons to reduce BMAA toxicity and in glia to enhance the toxicity of BMAA in flies.

## Introduction

Autosomal-dominant mutations in the Cu/Zn-superoxide dismutase 1 ( *sod1*) gene cause ~20% familial amyotrophic lateral sclerosis (fALS) ^1^. To date, more than 140 mutations linked to fALS have been identified in *sod1*. Some of these mutants are enzymatically functional ^2^, consistent with the idea that mutant *sod1* results in a toxic gain-of-function rather than the loss of SOD1 activity ^3^. This is further supported by studies of a mouse model where deletion of *sod1* does not cause ALS-like motoneuron disease ^4, 5^.

Besides motoneurons, glial cells have also been known to play a role in SOD1-linked ALS. Using chimeric animals, Clement and colleagues showed that mutant SOD1 expressed in motoneurons survived significantly longer and delayed degeneration if the neighboring cells did not express mutant SOD1 ^6^. Other investigators demonstrated that wild type MN neighbored with microglia and macrophages expressing mutant SOD1 underwent degeneration and contained ubiquitin-positive inclusions ^7, 8^. Yamanaka and colleagues showed that expression of wild type SOD1 outside of MN delayed the onset of the disease and extended the lifespan of transgenic mice up to 50% ^9^. These experiments support the notion that fALS is a non-cell autonomous disease process where glial cells play a critical role in the progression of ALS.

Several hypotheses have been proposed to account for non-cell autonomous mechanisms of ALS. Loss of the excitatory amino acid glutamate transporter (EAAT2) in astrocytes may cause glutamate-dependent excitotoxicity promoting disease progression ^10^. Higher inflammatory response from microglia mediated by mutant-expressing astrocytes ^11^ or inability to regulate glutamate receptor subunit 2 expression in motoneurons by mutant astrocytes ^12^ have also been suggested as mechanisms for ALS development. Finally, release of unknown toxins by astrocytes as evidenced by rapid death of embryonic stem cell-derived motoneurons when co-cultured with mutant expressing glial cells also has been suggested ^13, 14^. These studies point to the importance of bilateral interactions between astrocytes and motor neurons in ALS development and progression.

In addition to genetic inheritance of fALS, environmental agents have also been identified as causes for ALS. β-N-methylamino-L-alanine (BMAA) is a non-specific amino acid that was found in the post-mortem brain samples of the people of Guam who developed amyotrophic lateral sclerosis/Parkinson-dementia complex (ALS/PDC) during the 1940s ^15, 16^. BMAA is generated by cyanobacteria and can be found in many aquatic and/or terrestrial ecosystems and is believed to accumulate in living organisms by a process called biomagnification ^17^. Ross and colleagues reported behavioral changes in mice in response to intracerebroventricularly administered BMAA; these mice were hyper-excitable followed by whole body shaking/wobbling ^18^. Intracranially injected BMAA caused injuries of hippocampal neurons in mice ^19^. Using a cell culture system derived from spinal cord tissues of 13-day old mouse embryos, Rao and colleagues suggested that BMAA was selectively toxic to motor neurons via the AMP/Kainate receptor ^20^. Lobner and colleagues have also proposed that BMAA is an agonist for NMDA and mGluR5 receptors because BMAA induces oxidative stress in cultured mouse cortical neurons ^21^.

Increasing oxidative stress and reactive oxygen species (ROS) either by the herbicide paraquat or by excessive levels of hydrogen peroxide may also enhance neurodegeneration ^22– 24^. Paraquat has been implicated in Parkinson’s disease ^22, 25, 26^ and in ALS. In a case-control study of ALS in northern Italy, Bonvicini and colleagues found that compared to age- and sex-matched controls, more ALS patients had experienced occupational pesticide exposure in excess of 6 months ^27^. There was an increased risk of ALS among employees of the Dow Chemical Company who were exposed to the herbicide 2,4-dichlorophenoxyacetic acid versus other Dow employees ^28^. Paraquat showed a decrease in ubiquitously expressed survival motor neuron 1 (SMN1) in the human cell line NSC34, implicating oxidative stress in the mechanism of ALS underlying SMN1 deficiency in familial ALS and potentially sporadic form of the disease ^29^. More recently, TAR DNA-binding protein 43 (TDP-43) has been implicated in the etiology of sporadic ALS ^30^. Using human neuron culture, Ayala *et al.* showed that H _2_O _2_ (10 µM) caused accumulation of TDP-43 in the cytosolic fractions, a condition presumably linked to pathology of ALS ^31^.

BMAA, paraquat, and hydrogen peroxide are also known to have debilitating effects on motor activity and longevity in fruit flies ^25, 32, 33^. Pesticides were shown to cause complete or partial chromosome loss in repair-defective female *Drosophila* ^34^. The effect of paraquat on longevity is best studied in fly models of Parkinson's disease ^35^. Flies mutant for DJ-1 showed striking sensitivity to agents such as paraquat and rotenone ^36^. Islam and colleagues have shown a significant loss of dopaminergic neurons when these flies were treated with either 500 µM rotenone or 10 mM paraquat ^37^. Flies mutant for the glial *lazarillo*, which codes for the homologue of human Apolipoprotein D, also showed significantly reduced lifespan under paraquat treatment ^38^. BMAA was found to be a glutamate agonist, reduced lifespan, impaired motor activity, and caused memory deficits in fruit flies ^39^. Mekdara and colleagues recently also have reported a BMAA-induced decline in locomotion in a dose-dependent manner in *Drosophila* ^40^. More recently, fruit flies fed with BMAA were shown to cause prolonged open state of the NMDA receptor channel ^41^, suggesting a conserved molecular pathway for BMAA toxicity from mammals to flies.

How BMAA, paraquat, and H _2_O _2_ interact with mutant SOD1, however, remains unclear. In this report, we aim to understand the roles of mutant SOD1 expressed in different cell types in fly longevity when these flies are exposed to BMAA, paraquat, and hydrogen peroxide. We overexpressed mutant SOD1 in a cell-type specific fashion in flies and then treated them with 3 mM BMAA (in 3% sucrose), 20 mM paraquat (in fly food), or 1% hydrogen peroxide (in 3% sucrose). All three chemicals shortened the lifespan of the flies. However, flies with MN expression of mutant SOD1 lived longer on BMAA-containing food compared to control flies. Interestingly, flies with glial expression of mutant SOD1 died sooner on BMAA. Different from BMAA, H _2_O _2_ reduced lifespan of flies further in flies with mutant SOD1 expressed in any of the three cell types compared to controls. Unlike BMAA and H _2_O _2_, paraquat reduced longevity to a similar extent in both control flies and flies expressing mutant or wildtype SOD1. Hence, our results reveal a novel role for glial mutant SOD1 in mediating BMAA toxicity and a surprising role for motor neuronal mutant SOD1 in resisting BMAA toxicity. Furthermore, mutant SOD1 enhances H _2_O _2_ toxicity in shortening fly lifespan.

## Materials and methods

### *Drosophila* Stocks and Transgenic Flies

All experimental flies were reared on cornmeal agar medium at constant room temperature (22°C) under a ~12 h/12 h light/dark cycle. Male flies were used in all experiments. F1 flies from Gal4 drivers>Canton S (CS) and Gal4 drivers>UAS-d *sod1* crosses were used as control strain in all studies. Each food vial housed 10 flies.

The human *sod1* transgenic lines (h *sod1* ^WT^, h *sod1* ^A4V^, and h *sod1* ^G85R^), the wild type *Drosophila SOD1* (d *sod1*), and motor neuron (D42) and glial (M1B) Gal4 drivers were described previously ^42^. For h *sod1* ^G85R^, four independent insertions were recombined to bring its expression level closer to that of h *sod1* ^wt^, and h *sod1* ^A4V^. The GAL4-UAS expression system was used to express *sod1* transgenes in particular cell types. For motoneuron-specific expression, the D42-Gal4 driver line was used ^43, 44^. To express *sod1* in glial cells alone M1B-Gal4 driver was used ^42^. To express *sod1* in both motoneurons and glial cells, both D42 and M1B Gal4 drivers were used.

### BMAA treatment

L-BMAA (B107) was purchased from Sigma (St. Louis, MO, USA) and dissolved in water. The BMAA solution was supplemented with 3% sucrose before treatment. Two pieces of 3M filter paper were placed in a clear plastic vial (2.5 × 9.5 cm ^2^) and soaked with droplets of the BMAA/sucrose solution. The flies were transferred to new vials with newly soaked filter paper every 24 hours. Care was taken to avoid excessive accumulation of solutions to prevent the drowning of flies. The control flies were treated with 1, 3, 5 and 10 mM BMAA. However, for all the experiments of the transgenic flies 3 mM BMAA was used. Five and 35 day-old flies with undamaged wings were selected for the BMAA treatment. Flies under treatment and their control groups were monitored for survival every 24 hours. A fly was considered dead when it did not show any body movement after a few gentle tapings at the vial. When necessary, a light microscope was used to determine any movements of the limbs after a few gentle taps to confirm a fly's death. The numbers of dead flies were recorded for each day. Changes in longevity were computed by comparing with D42>UAS-d *sod1* and D42>+ lines.

### Paraquat treatment

Male *sod1* transgenic and wild type flies aged 1–3 days were fed with standard cornmeal agar food containing 20 mM paraquat. The number of dead flies was recorded every 24 hours and the live flies were transferred to corresponding new vials with food and paraquat. The flies were observed until all flies were dead in experimental groups.

### Hydrogen peroxide treatment

30% hydrogen peroxide (EMD Chemicals, HX0635, Darmstadt, Germany) was diluted to 1% with a 3% sucrose solution. Approximately 100 male flies in groups of ten were aged to 5 days on standard cornmeal agar. They were then starved for six hours in clean plastic vials containing two pieces of filter paper soaked with 300µl of water. After 6 hours, starved flies were transferred to fresh vials containing filter paper soaked with either 300µl of the 1% H _2_O _2_/sucrose solution or 300µl of 3% sucrose solution as a control. Every 24 hours, the flies were transferred to fresh vials with newly soaked paper and monitored for survival. Flies were maintained at 22°C under a 12h/12h light/dark cycle for the duration of the experiment.

### Climbing assay

We modified the techniques of measuring motor activity from a previously published method ^45^. Briefly, 59 day-old 10 male flies without any visible physical defect were selected and transferred to a vial with fresh food and harvested overnight (n=50 per genotype). These flies were not rendered to BMAA treatment. Next day, in a quiet area of the lab with normal light level, 10 flies were transferred to a clean and dry 100 ml glass cylinder. The flies were gently tapped down to the bottom of the cylinder and monitored for their climbing behavior in a 20-second period. These flies were assigned into three groups. Group 1: flies able to climb past 20 ml (35 mm); group 2: flies still climbing or roaming around in the bin in between 5 ml and 20 ml (7.5 mm -35 mm); group 3: flies that could not climb past 5 ml (7.5 mm).

### Experiment replication and statistics

All data were obtained from at least 3 experiments. All *p* values were based on 2-tailed t-tests or One-way ANOVA tests in the Prizm software and differences were considered significant if p<0.05. Error bars represent standard errors of the mean (SEM).

## Results

### Motor activity of the SOD1 transgenic flies is altered by mutant SOD1 in a cell-specific fashion

**Figure 1.**
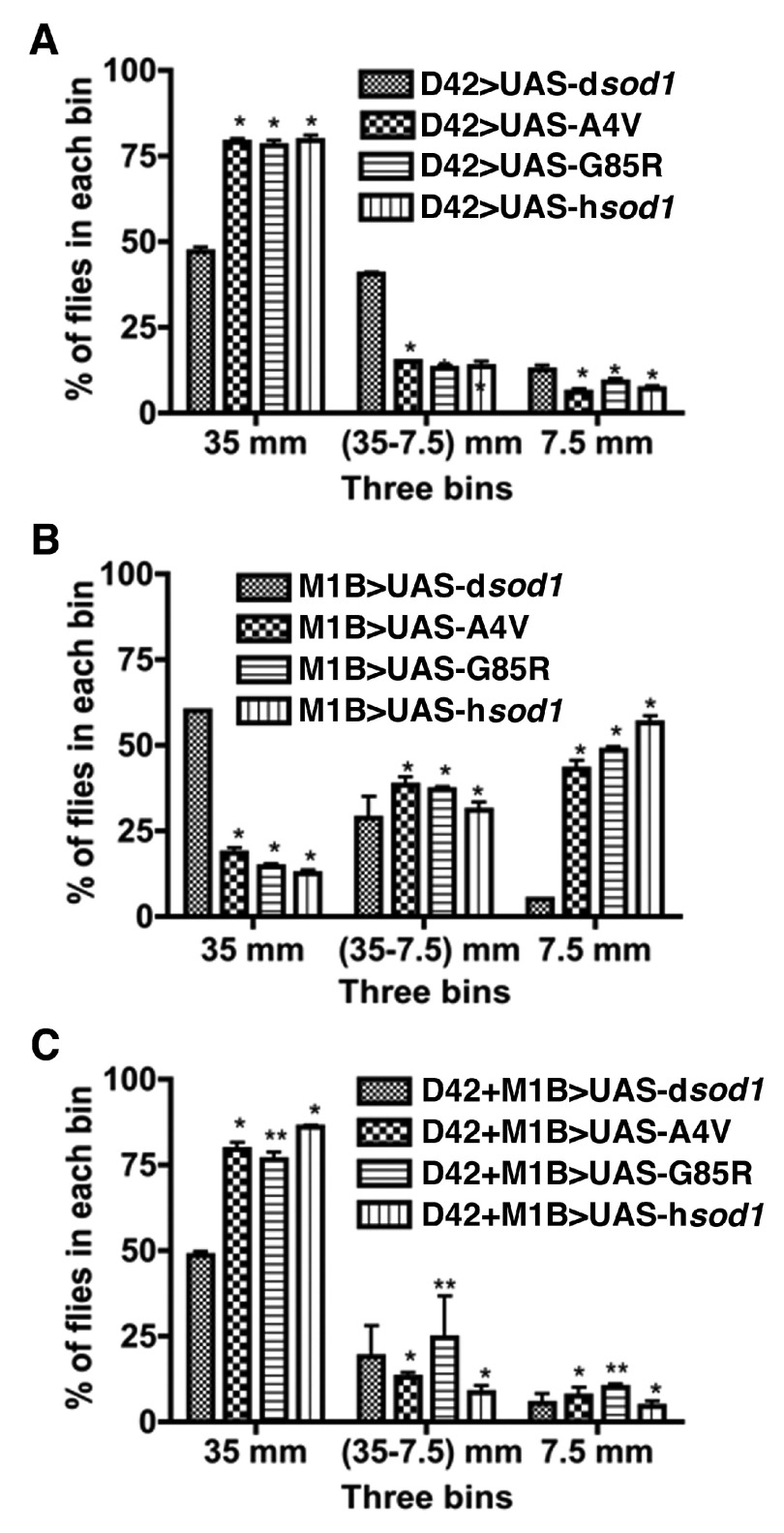
Climbing behavior of 60 day-old flies. Healthy looking 60 day-old male flies were selected for this assay. Ten flies were placed in a 100 ml glass cylinder. Following a brief and gentle tapping, the distribution of flies was then counted in 3 bins at different heights (above or at 35 mm height, between 7.5 mm and 35 mm, and 7.5 mm or lower). Climbing pattern of flies expressing SOD1 in motoneurons ( **A**), glial cells ( **B**), and co-expressing SOD1 in both motoneurons and glial cells ( **C**) are shown. Flies expressing human SOD1 proteins in glial cells show a significant shift of distribution to the bottom of the cylinder compared to M1B>UAS-d *sod1* (the control fly) and in comparison with their expression in motoneurons ( **A**) or dual expression in MNs + glia ( **C**). These observations suggest a significant impairment of climbing activities at 60 days in flies expressing human SOD1s in glia. The statistical significances were calculated using Prizm software, Two-way ANOVA and * p<0.01 and ** p<0.03 (n=50 for each genotype).

Climbing is an innate negative gravitaxic behavior of *Drosophila*. Climbing activities have been used to measure the motor functions of fruit flies ^45^. Transgenic mice overexpressing SOD1 showed late-onset progressive motor defects ^46^. In flies, expression of mutant SOD1 reduced climbing capability ^42^. Here, we tested the effect of mutant SOD1 overexpression in MN, glia, or in both on fly climbing and noticed a remarkable locomotive slowdown as the flies approached around 60 days. In a modified climbing assay, we assigned all our experimental flies a bin in a 100 ml cylinder depending on their location during a 20 second climbing trip. The fastest climber reached the top bin (above 35 mm), the sluggish group reached the middle bin (above 7.5 mm but below 35 mm), and the slowest group remained at the bottom bin (below 7.5 mm). We found that about 80% of the D42>UAS- *sod1* flies reached the top bin ( Figure 1A). On the contrary, less than 20% of the M1B>UAS- *sod1* flies reached the top bin ( Figure 1B). Overall, there is a significant shift of fly distributions to the bottom and middle portions of the graduated cylinder in M1B>UAS- *sod1* flies. Interestingly, however, about 80% of the D42+M1B>UAS- *sod1* flies also reached the top bin ( Figure 1C). These results indicate significantly differential effects of SOD1 proteins in motor neurons and glial cells; mutant SOD1 in glia has a more profound negative effect on climbing in aging flies.

Climbing behavior of 60 day-old flies. Healthy looking 60 day-old male flies were selected for this assay. Ten flies were placed in a 100 ml glass cylinder. Following a brief and gentle tapping, the distribution of flies was then counted in 3 bins at different heights (above or at 35 mm height, between 7.5 mm and 35 mm, and 7.5 mm or lower). Climbing pattern of flies expressing SOD1 in motoneurons (A), glial cells (B), and co-expressing SOD1 in both motoneurons and glial cells (C) are shown. Flies expressing human SOD1 proteins in glial cells show a significant shift of distribution to the bottom of the cylinder compared to M1B>UAS-dsod1 (the control fly) and in comparison with their expression in motoneurons (A) or dual expression in MNs + glia (C). These observations suggest a significant impairment of climbing activities at 60 days in flies expressing human SOD1s in glia. The statistical significances were calculated using Prizm software, Two-way ANOVA and * p

### BMAA reduces lifespan of wild-type flies in a dose-dependent manner

**Figure 2.**
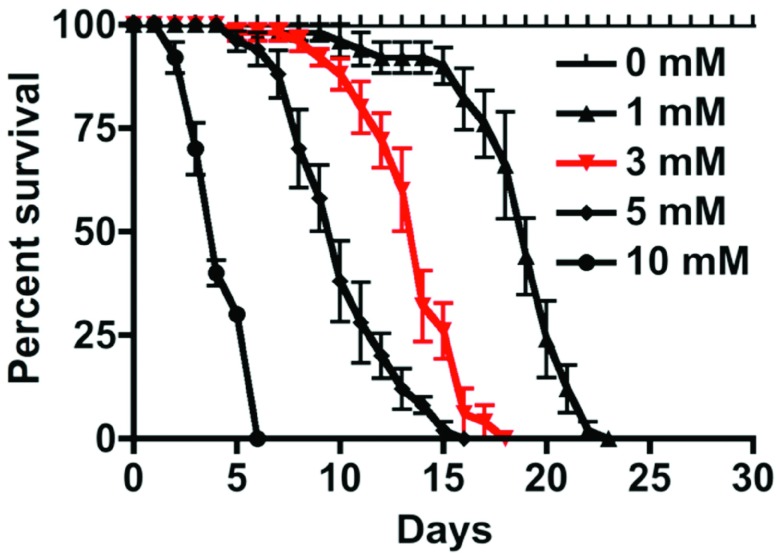
Survival curve of wild type ( *Canton S*) *Drosophila* under various dosages of BMAA. Five day-old male flies were reared in 1, 3, 5, and 10 mM BMAA diluted in 3% sucrose for up to 30 days (10 flies per vial). The 50% survival time for 1, 3, 5, and 10 mM BMAA was 19, 14, 10, and 4 days, respectively. The sham (0 mM BMAA) treated flies did not show any toxicity during the observation period. The statistics was performed using One-way ANOVA analysis of variance in Prizm software, p<0.01 (n=50 for 1, 3, and 5 mM BMAA groups; n=20 for the 0 mM BMAA group).

Several reports have shown neurotoxic effects of BMAA in animal models ^15, 18^. More recently, Zhou and colleagues studied the dietary effects of BMAA in *Drosophila* ^32^ and found BMAA as the most toxic among a few other excitatory amino acids to 1–3 day-old flies. BMAA shortened lifespan and resulted in neurological deficiencies. To examine the age-dependent effects of BMAA on longevity of flies, we fed 5 day-old wild type (Canton S) flies with 1, 3, 5 and 10 mM BMAA ( Figure 2) and noticed a dosage-dependent decline in survival in both age groups. We opted to use 3 mM concentration in all subsequent experiments because of its intermediate effects on longevity.

Survival curve of wild type (Canton S) Drosophila under various dosages of BMAA. Five day-old male flies were reared in 1, 3, 5, and 10 mM BMAA diluted in 3% sucrose for up to 30 days (10 flies per vial). The 50% survival time for 1, 3, 5, and 10 mM BMAA was 19, 14, 10, and 4 days, respectively. The sham (0 mM BMAA) treated flies did not show any toxicity during the observation period. The statistics was performed using One-way ANOVA analysis of variance in Prizm software, p

### Effects of BMAA on *SOD1* transgenic flies are cell-type specific

**Figure 3.**
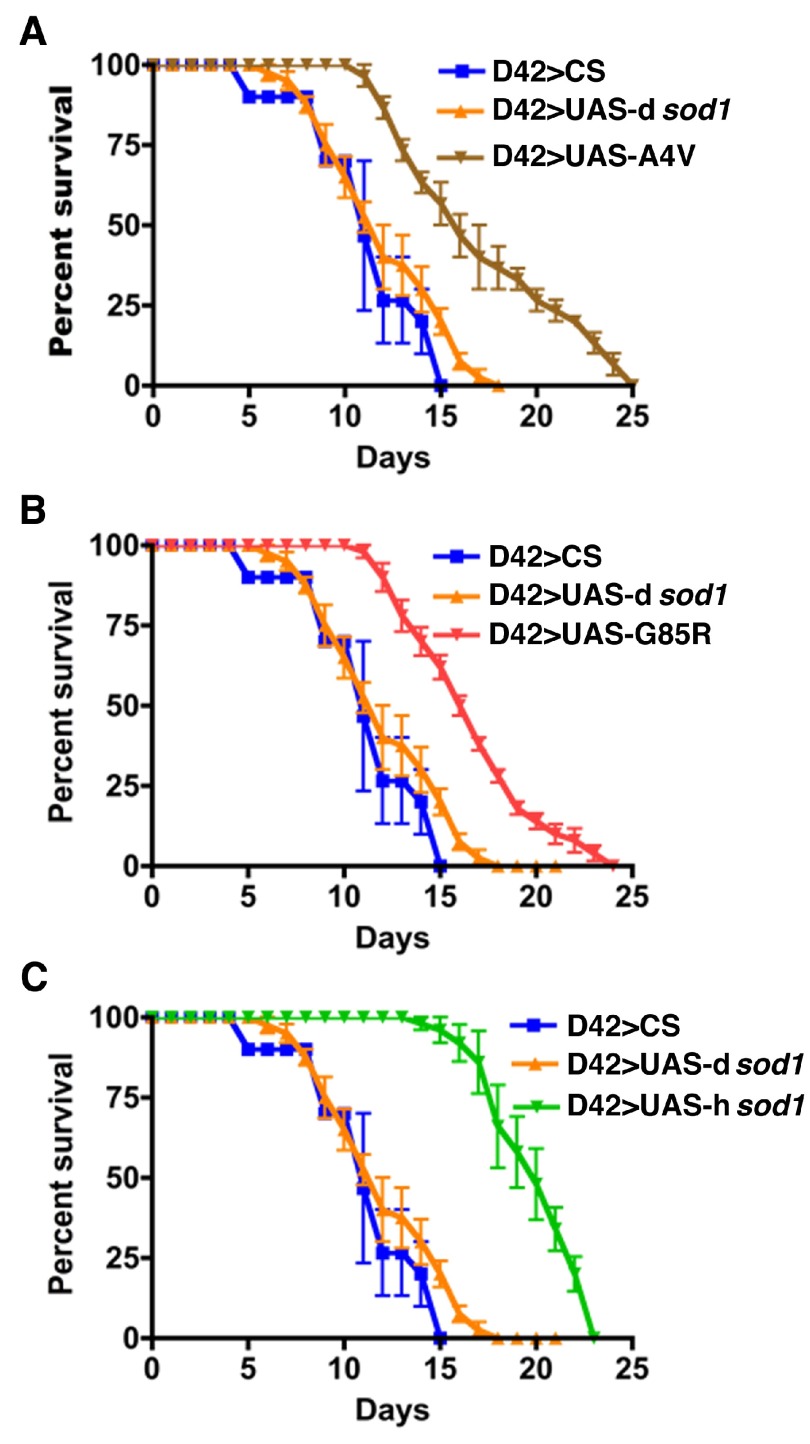
Human SOD1 overexpressed in motoneurons confers resistance to BMAA treatment. Shown are survival rates of 5 day-old male *sod1* transgenic flies under 3 mM BMAA treatment (10 flies per vial). Panels A, B, and C represent the survival rate of flies expressing mutant human A4V, G85R, wild type SOD1 proteins and control flies (D42>CS and D42>UAS-d *sod1*) in motoneurons using the D42-Gal4 driver. Flies expressing the human SOD1s (both the hSOD1 ^WT^, and mutants, hSOD1 ^A4V^ and hSOD1 ^G85R^) survived longer compared to the control flies. At the 50% survival rate, the longevity is increased by 33%, 41% and 66% for A4V, G85R, wt SOD1, respectively, when compared to D42>d *sod1* and D42>CS flies. Differences were considered statistically significant if p<0.05 and were calculated by Paired t-test method by using Prizm software. n=30 for D42>CS and D42>A4V, respectively; n=50 for the rest of genotypes.

To determine a cellular target of SOD1 and its interaction with BMAA, H _2_O _2_, and paraquat we used the GAL4-UAS system to express SOD1 proteins in specific neuronal or glial cell types. We used D42-Gal4 ^43, 44^, M1B-Gal4 and a recombined D42+M1B-Gal4 drivers to express wild type human *sod1* (h *sod1* ^wt^) and two fALS-linked mutants of h *sod1* (h *sod1* ^A4V^, h *sod1* ^G85R^) in motoneurons (D42>UAS-h *sod1*), glial cells (M1B>UAS-h *sod1*), and in both motoneurons and glial cells (D42+M1B>UAS-h *sod1*), respectively. The *Drosophila* native *sod1* (driver>UAS-d *sod1*) and wild type (driver>CS) flies were also crossed to each driver and included as controls in all experiments. The male F1 flies were collected on day 1 after they emerged from the pupal case and 10 flies were housed per vial. These flies were fed with 3 mM BMAA in 3% sucrose solution at the age of 5 and 35 days for up to 30 days. The flies under treatment were monitored every 24 hours and the numbers of dead flies were recorded. The surviving flies were transferred to new food vials after every count. Our results show that the lifespan for 50% population alive (L50) for D42>UAS-h *sod1* ^A4V^, D42>UAS-h *sod1* ^G85R^, D42>UAS-h *sod1* ^wt^ flies was increased 33%, 41%, and 66%, respectively, when compared with the D42>UAS-d *sod1* flies ( Figure 3 A–C, and Table 1). Comparison of the same transgenic flies with the D42>CS flies treated with 3 mM BMAA also produced an increase in survival of the all D42>UAS-h *sod1* flies ( Figure 3).

Human SOD1 overexpressed in motoneurons confers resistance to BMAA treatment. Shown are survival rates of 5 day-old male sod1 transgenic flies under 3 mM BMAA treatment (10 flies per vial). Panels A, B, and C represent the survival rate of flies expressing mutant human A4V, G85R, wild type SOD1 proteins and control flies (D42>CS and D42>UAS-dsod1) in motoneurons using the D42-Gal4 driver. Flies expressing the human SOD1s (both the hSOD1WT, and mutants, hSOD1A4V and hSOD1G85R) survived longer compared to the control flies. At the 50% survival rate, the longevity is increased by 33%, 41% and 66 % for A4V, G85R, wt SOD1, respectively, when compared to D42>dsod1 and D42>CS flies. Differences were considered statistically significant if pCS and D42>A4V, respectively; n=50 for the rest of genotypes.

**Table 1.** Changes in survival for 50% of fly population under 3 mM BMAA treatment (%).

	D42		M1B		D42+M1B	
Age	5 days	35 days	5 days	35 days	5 days	35 days
A4V	33	37	-19	-31	0	8
G85R	41	30	-19	-38	17	30
h *sod1*	66	30	-28	-38	22	15

A minus (‘-’) indicates a decrease in survival rate.

**Figure 4.**
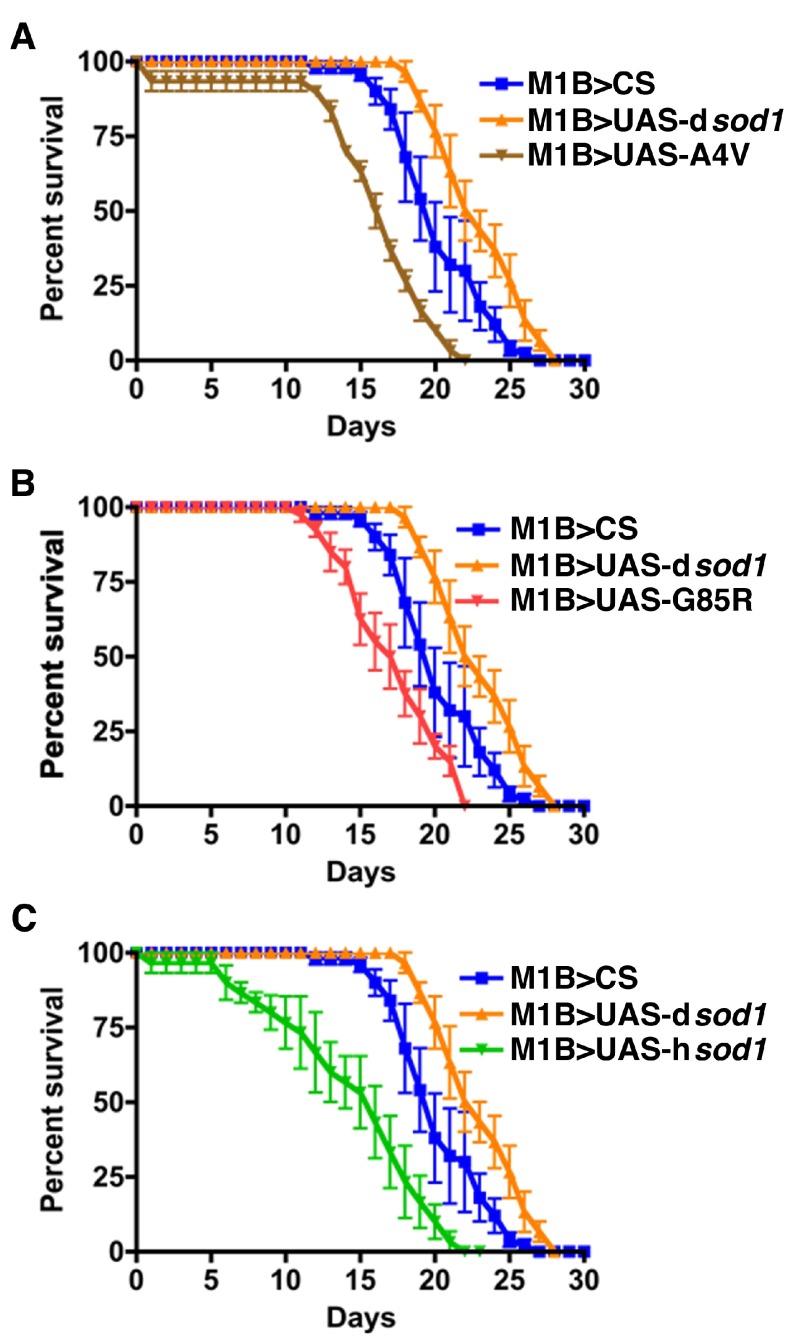
Human SOD1 overexpressed in glial cells accelerates fly death by BMAA. Shown are survival rates of 5 day-old male *sod1* transgenic flies under 3 mM BMAA treatment (10 flies per vial). Panels A, B, and C represent flies expressing mutant human A4V, G85R, wild type SOD1 proteins, respectively, in comparison with control flies (M1B>CS and M1B>UAS-d *sod1*) in glia using the M1B-Gal4 driver. At the 50% survival rate, the longevity for A4V, G85R, and hSOD1 is reduced by 19%, 19%, 28%, respectively, compared to D42>UAS-d *sod1* and D42>CS. Differences were considered statistically significant if p<0.05 and were calculated by a paired t-test method using Prizm software. n=30 for M1B>A4V and M1B>d *sod1*, respectively; n=40 for M1B>G85R; n=50 for M1B>CS.

**Figure 5.**
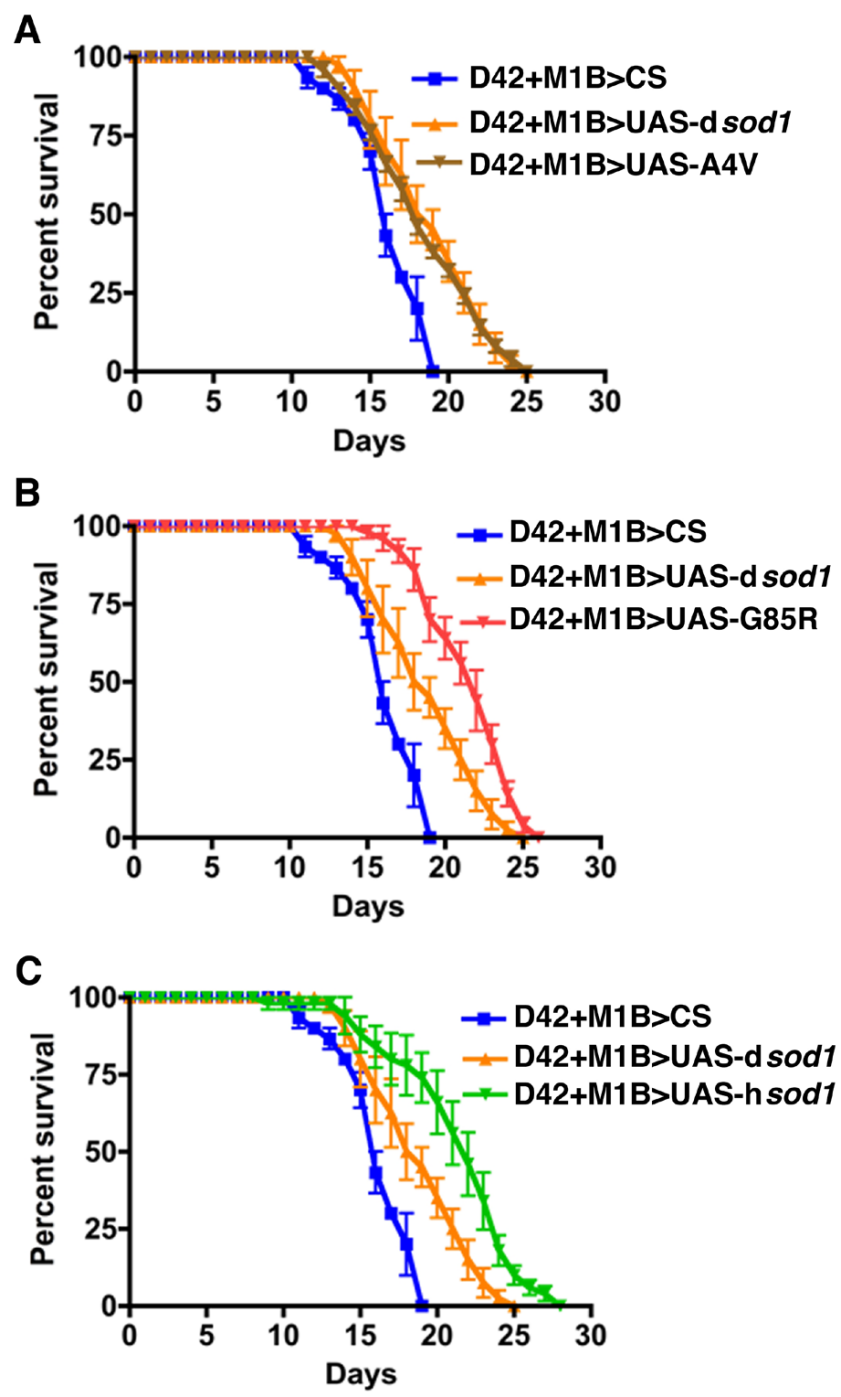
Human SOD1 co-overexpressed in motoneurons and glial cells and treated with 3 mM BMAA. Shown are survival rates of 5 day-old male *sod1* transgenic flies (10 flies per vial). Panels A, B, and C represent flies expressing mutant human A4V, G85R, and wild type SOD1 proteins and control flies (D42+M1B>CS and D42+M1B>UAS-d *sod1*) in motoneurons and glia using D42-Gal4, M1B-Gal4, respectively. At the 50% survival rate, the longevity for A4V, G85R, and hSOD1 is increased by 0%, 17%, and 22% for A4V, G85R, and hSOD1 flies, respectively, compared to D42>UAS-d *sod1* and D42>CS. Differences were considered statistically significant if p<0.05 and were calculated by a paired t-test method using Prizm software (n=50 for each genotype).

**Figure S1.**
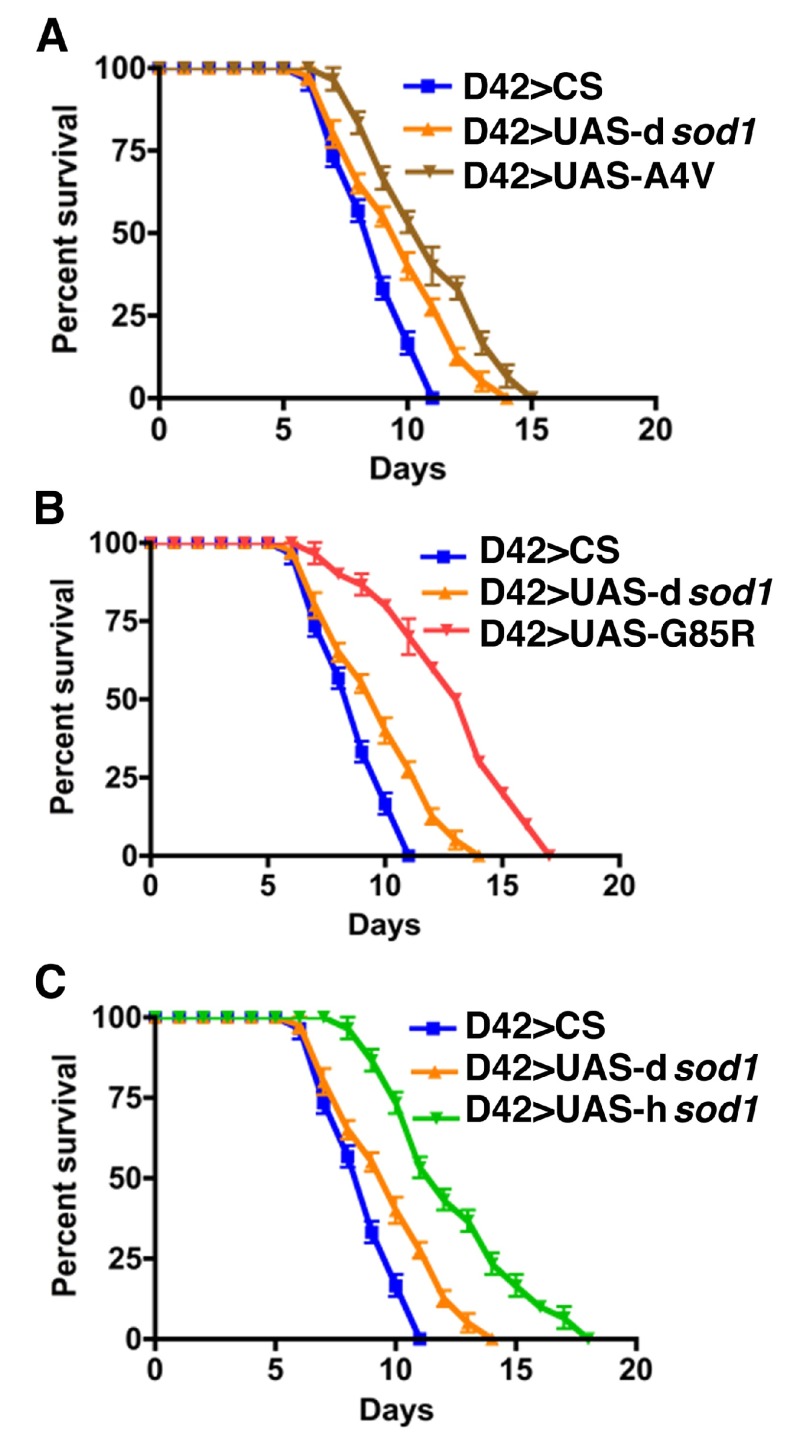
Human SOD1 over-expressed in motor neurons confers resistance to BMAA treatment. Shown are survival rates of 35 day-old male *sod1* transgenic flies under 3 mM BMAA treatment (10 flies per vial). Panels A, B, and C represent flies expressing mutant human A4V, G85R, and wild type SOD1 proteins, respectively. Like 5 day-old time points ( Figure 3 A–C), the SOD1 flies survived longer compared to the controls (CS and dSOD1). The 50% survival rate is increased by 10, 30 and 30% for A4V, G85R, wt SOD1, respectively, when compared to dSOD1 flies. Differences were considered statistically significant if p<0.01 and were calculated by a paired t-Test method by using Prizm software (n=30 for each genotype).

On the contrary, the M1B>UAS-h *sod1* flies were more sensitive to BMAA insult. The L50 for these flies was reduced by 19%, 30%, and 30% for M1B>UAS-h *sod1* ^A4V^, M1B>UAS-h *sod1* ^G85R^, M1B>UAS-h *sod1* ^wt^, respectively compared to M1B>UAS-d *sod1* flies ( Figure 4 A, B, C and Table 1). However, to our surprise, the D42+M1B>UAS-h *sod1* flies were found to be also capable of resisting BMAA toxicity in contrast to M1B>UAS-h *sod1* flies. The resistance in this group was not as strong as it was in the D42>UAS-h *sod1* group. The results show that the L50 for the D42+M1B>UAS-h *sod1* flies was increased to 0%, 17%, and 22% for hSOD1 ^A4V^, hSOD1 ^G85R^, hSOD1 ^wt^, respectively in comparison with D42+M1B>UAS-d *sod1* flies ( Figure 5 A, B, C and Table 1). The analysis of the results from the 35 day-old flies showed that the extension or reduction in survival rates was consistent genotype-wide and in agreement with those found in 5 days old flies ( Table 1 and Figure S1– Figure S3).

Human SOD1 overexpressed in glial cells accelerates fly death by BMAA. Shown are survival rates of 35 day-old male sod1 transgenic flies under 3 mM BMAA treatment. Panels A, B, and C represent flies expressing mutant human A4V, G85R, and wild type SOD1 proteins, respectively. Like 5 day-old time points (Figure 3, A-C), the SOD1 flies survived longer compared to the controls (CS and dSOD1). The 50% survival rate is increased by 10, 30 and 30% for A4V, G85R, wt SOD1, respectively, when compared to dSOD1 flies. Differences were considered statistically significant if p

Human SOD1 co-overexpressed in motoneurons and glial cells and treated with 3 mM BMAA. Shown are survival rates of 35 day-old male sod1 transgenic flies under 3 mM BMAA treatment. Panels A, B, and C represent flies expressing mutant human A4V, G85R, and wild type SOD1 proteins, respectively. Like 5 day-old time points (Figure 3, A-C), the SOD1 flies survived longer compared to the controls (CS and dSOD1). The 50% survival rate is increased by 10, 30 and 30% for A4V, G85R, wt SOD1, respectively, when compared to dSOD1 flies. Differences were considered statistically significant if p

Human SOD1 over-expressed in motor neurons confers resistance to BMAA treatment. Shown are survival rates of 35 day-old male sod1 transgenic flies under 3 mM BMAA treatment (10 flies per vial). Panels A, B, and C represent flies expressing mutant human A4V, G85R, and wild type SOD1 proteins, respectively. Like 5 day-old time points (Figure 3, A-C), the SOD1 flies survived longer compared to the controls (CS and dSOD1). The 50% survival rate is increased by 10, 30 and 30% for A4V, G85R, wt SOD1, respectively, when compared to dSOD1 flies. Differences were considered statistically significant if p

**Figure S2.**
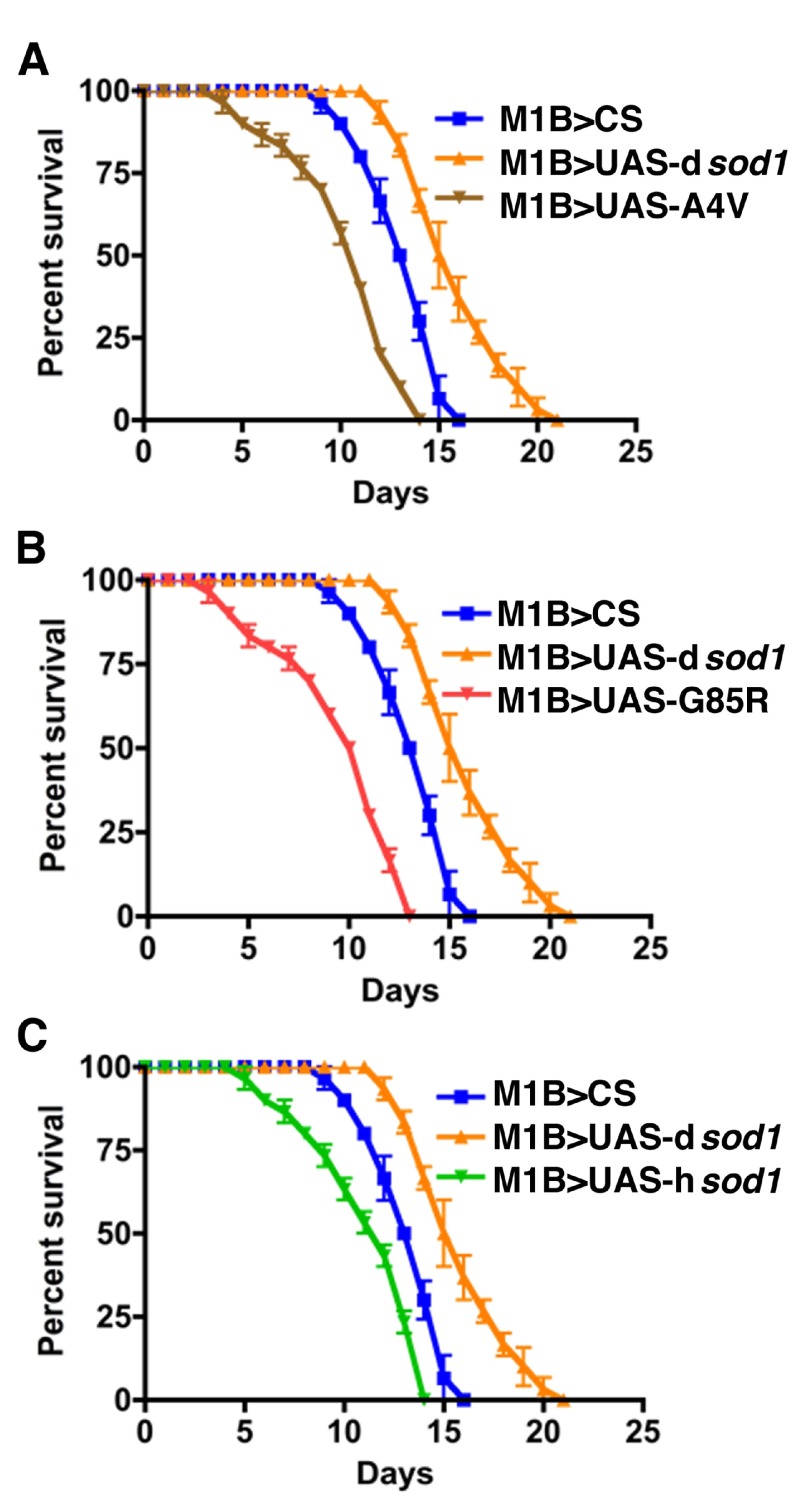
Human SOD1 over-expressed in glial cells enhances the effect of BMAA on reducing the longevity of flies. Shown are survival rates of 35 day-old male *sod1* transgenic flies under 3 mM BMAA treatment (10 flies per vial). Panels A, B, and C represent flies expressing mutant human A4V, G85R, wild type SOD1 proteins. The 50% survival rate of A4V, G85R, and hSOD1 when compared with dSOD1 is decreased by 31, 38, and 38%, respectively. Differences were considered statistically significant if p<0.02 and were calculated by a paired t-test method by using Prizm software (n=30 for each genotype).

Human SOD1 over-expressed in glial cells enhances the effect of BMAA on reducing the longevity of flies. Shown are survival rates of 35 day-old male sod1 transgenic flies under 3 mM BMAA treatment (10 flies per vial). Panels A, B, and C represent flies expressing mutant human A4V, G85R, wild type SOD1 proteins. The 50% survival rate of A4V, G85R, and hSOD1 when compared with dSOD1 is decreased by 31, 38, and 38%, respectively. Differences were considered statistically significant if p

**Figure S3.**
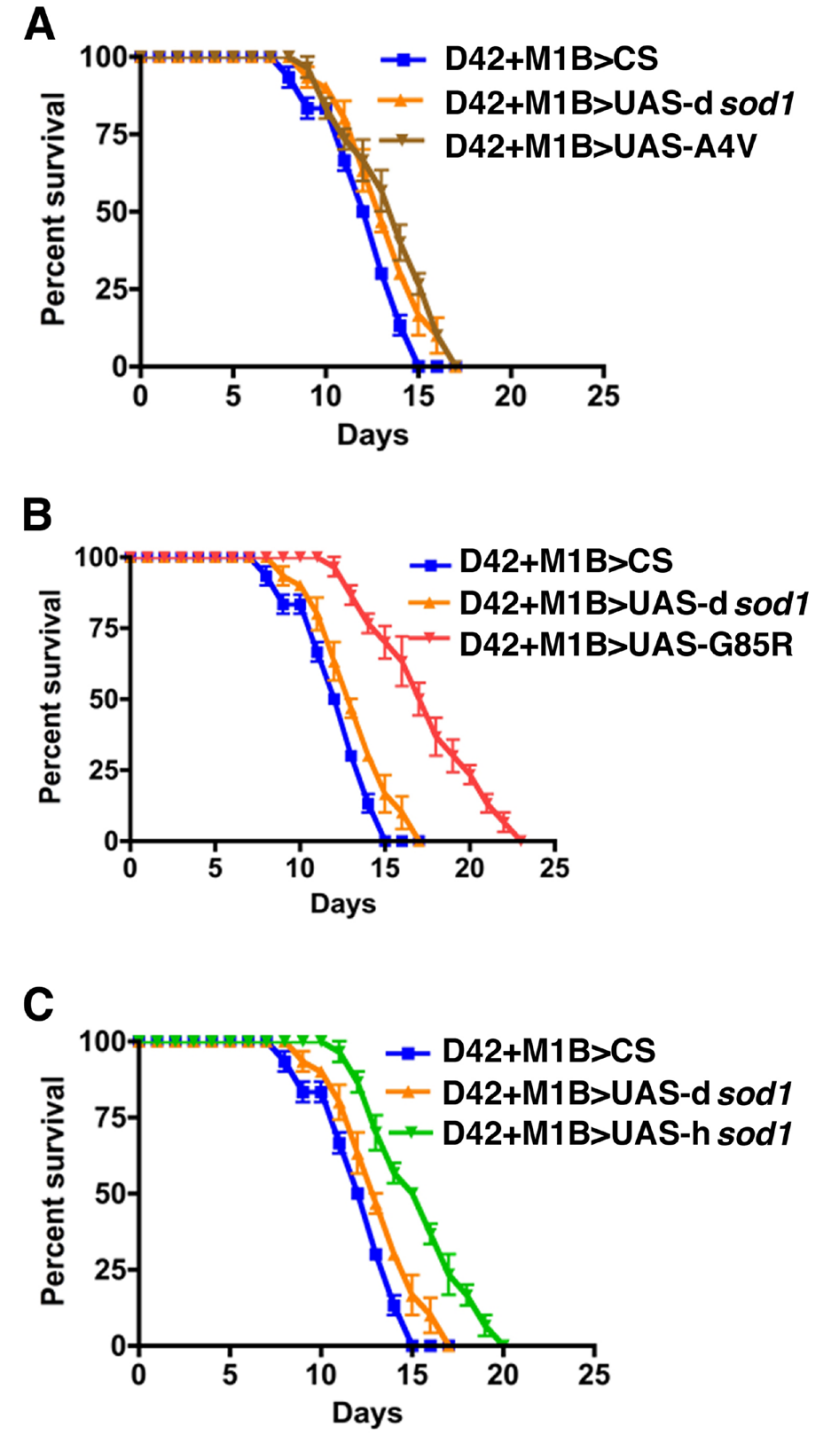
Human SOD1 co-overexpressed in motor neurons and glial cells and treated with 3 mM BMAA. Shown are survival rates of 35 day-old male *sod1* transgenic flies under 3 mM BMAA treatment (10 flies per vial). Panels A, B and C represent flies expressing mutant human A4V, G85R, wild type SOD1 proteins. The 50% survival rate of A4V, G85R, and hSOD1 when compared with dSOD1 is increased by 8, 30, and 15%, respectively. Differences were considered statistically significant if p<0.01 and were calculated by a paired t-test method using Prizm software (n=30 for each genotype).

Human SOD1 co-overexpressed in motor neurons and glial cells and treated with 3 mM BMAA. Shown are survival rates of 35 day-old male sod1 transgenic flies under 3 mM BMAA treatment (10 flies per vial). Panels A, B and C represent flies expressing mutant human A4V, G85R, wild type SOD1 proteins. The 50% survival rate of A4V, G85R, and hSOD1 when compared with dSOD1 is increased by 8, 30, and 15%, respectively. Differences were considered statistically significant if p

### Mutant SOD1 exacerbates the effects of hydrogen peroxide on fly lifespan

**Figure 6.**
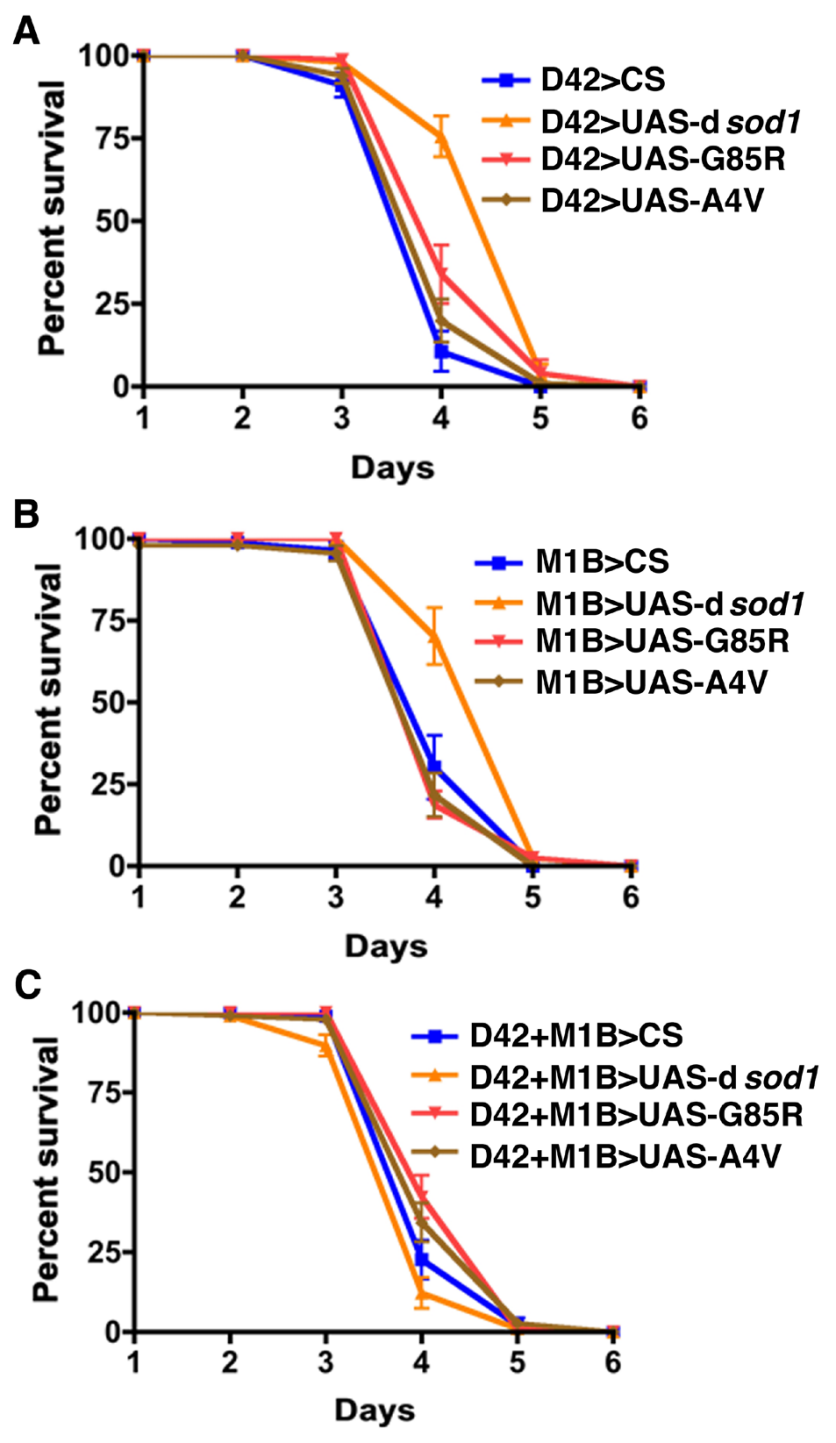
Effects of H _2_O _2_ on SOD1 transgenic flies. Five day-old male flies expressing SOD1 proteins in motor neurons ( **A**), glial cells ( **B**) and in motoneurons together with glial cells ( **C**) were treated with 1% H _2_O _2_diluted in 3% sucrose. The results indicate an enhancing effect of H _2_O _2_ on mutant SOD1 toxiciy in MNs or glia when compared to driver>UAS-d *sod1*, but not to driver>CS control. Statistical analysis was performed using a two-tailed ANOVA method in Prizm software and was considered significant if p<0.05 (n=80–96 for each genotype).

Our study feeding flies with H _2_O _2_ (1% in 3% sucrose) confirms previous published results on longevity ^33^. In control flies (D42>+ or D42>UAS-d *sod1*), most flies survived the first day on H _2_O _2_, but gradually died within the next three-six days. We then tested whether mutant SOD1 expressed in MN or glia has any additional effects together with H _2_O _2_ on fly longevity. Our results show that all 5 day old flies began to die faster starting on the third day of treatment except D42>UAS-d *sod1*, and continued dying rapidly on day 4. On day 5, almost all flies, including D42>UAS-d *sod1*, were dead despite some resistance of dSOD1 on day 4 ( Figure 6A). When mutant SOD1 was expressed in glia alone, we observed a similar survival pattern as of D42>UAS-d *sod1* flies ( Figure 6B). In flies with dual expression of mutant SOD1 in both MNs and glia, the subtle resistance by the dSOD1 flies observed in D42 and M1B flies diminished and they were dead sooner than other flies. However, on day 5 all flies were dead ( Figure 6C). These results show that while human mutant SOD1 renders some resistance when expressed in either motor neurons alone or along with glia, these proteins adversely affect flies' survival when expressed in glial cells alone. Interestingly, though such resistance is very short in effect, *Drosophila* native SOD1 resists H _2_O _2_ toxicity in D42 and M1B flies, but not in D42+M1B flies. We also noted that our other control flies (Gal4 drivers>CS) were mostly similar to flies expressing mutant hSOD1. This makes it more difficult to interpret the data. The genetic argument is that dSOD1 would be a better control. Thus, we opted to consider the effects of mutant hSOD1s in comparison with dSOD1.

Effects of H2O2 on SOD1 transgenic flies. Five day-old male flies expressing SOD1 proteins in motor neurons (A), glial cells (B) and in motoneurons together with glial cells (C) were treated with 1% H2O2 diluted in 3% sucrose. The results indicate an enhancing effect of H2O2 on mutant SOD1 toxiciy in MNs or glia when compared to driver>UAS-dsod1, but not to driver>CS control. Statistical analysis was performed using a two-tailed ANOVA method in Prizm software and was considered significant if p

### Paraquat shortens the lifespan of flies independent of mutant SOD1 expression

**Figure S4.**
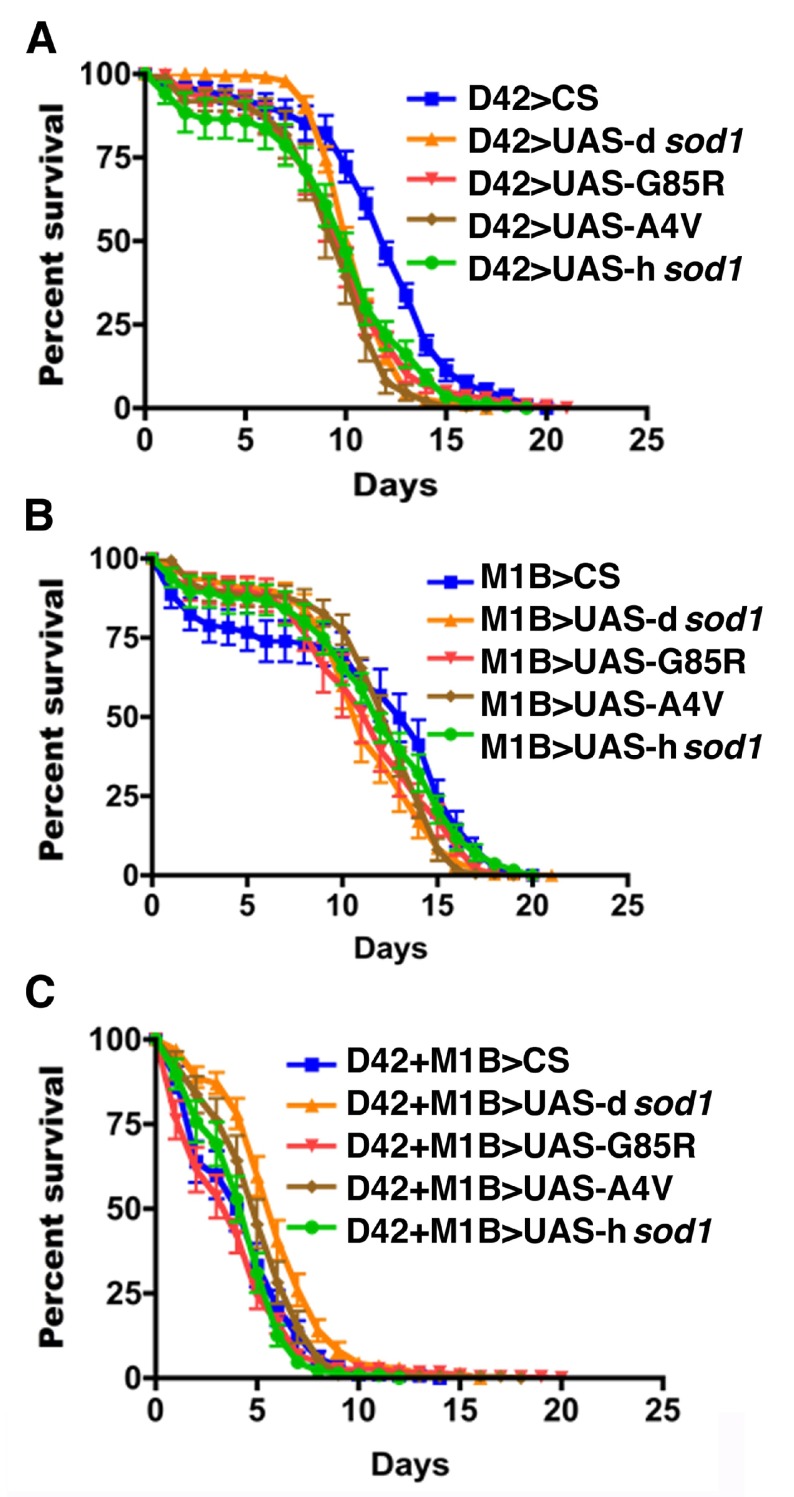
Effects of paraquat on SOD1 transgenic flies. Five day-old male flies expressing SOD1 proteins in motor neurons ( **A**), glial cells ( **B**) and in motoneurons together with glial cells ( **C**) were treated with 20 mM paraquat in fly food (10 flies per vial). The results do not indicate any significant effect of paraquat in SOD1 flies of all genotypes. Total flies uses for D42Gal4>CS, 183; D42Gal4>UAS- *dsod1*, 194; D42Gal4>UAS-hSOD1 ^A4V^, 138; D42Gal4>UAS-hSOD1 ^G85R^, 206; D42Gal4>UAS-hSOD1, 231. M1BGal4>CS, 148; M1BGal4>UAS- *dsod1*, 171; M1BGal4>UAS-hSOD1 ^A4V^, 164; M1BGal4>UAS-hSOD1 ^G85R^, 144; M1BGal4>UAS-hSOD1, 200. D42+M1BGal4>CS, 148; D42+M1BGal4>UAS- *dsod1*, 156; D42+M1BGal4>UAS-hSOD1 ^A4V^, 177; D42+M1BGal4>UAS-hSOD1 ^G85R^, 286; D42+M1BGal4>UAS-hSOD1, 213.

Paraquat is also known to shorten the lifespan of flies ^25, 33^. In a human cell line, NSC3, paraquat decreases expression of ubiquitously expressed Survival motor neuron resulting in increased oxidation ^29^. In our laboratory, we reproduced the shortening of lifespan in wild type flies under treatment with parquat. Interestingly, however, in contrast to BMAA, we observed no significant effects of paraquat (20 mM paraquat contained in fly food) on the lifespan of the SOD1-expressing flies compared to the wild type control groups ( Figure S4). The L50 was 11–12 days for D42>UAS- *sod1*, M1B>UAS- *sod1*, respectively, and L50 was 4–6 days for D42+M1B>UAS- *sod1* in the paraquat experiment.

Effects of paraquat on SOD1 transgenic flies. Five day-old male flies expressing SOD1 proteins in motor neurons (A), glial cells (B) and in motoneurons together with glial cells (C) were treated with 20 mM paraquat in fly food (10 flies per vial). The results do not indicate any significant effect of paraquat in SOD1 flies of all genotypes. Total flies uses for D42Gal4>CS, 183; D42Gal4>UAS-dsod1, 194; D42Gal4>UAS-hSOD1A4V, 138; D42Gal4>UAS-hSOD1G85R, 206; D42Gal4>UAS-hSOD1, 231. M1BGal4>CS, 148; M1BGal4>UAS-dsod1, 171; M1BGal4>UAS-hSOD1A4V, 164; M1BGal4>UAS-hSOD1G85R, 144; M1BGal4>UAS-hSOD1, 200. D42+M1BGal4>CS, 148; D42+M1BGal4>UAS-dsod1, 156; D42+M1BGal4>UAS-hSOD1A4V, 177; D42+M1BGal4>UAS-hSOD1G85R, 286; D42+M1BGal4>UAS-hSOD1, 213.

## Discussion

In this report we investigated the effects of BMAA, hydrogen peroxide, and paraquat on the lifespan of fruit flies and their interactions with mutant hSOD1 in search for a cell type-specific target for SOD1. As expected, all these chemicals significantly shortened the lifespan of wild-type and transgenic flies. However, significant reductions in lifespan of transgenic flies are cell type specific only for BMAA. In our studies, we added paraquat and hydrogen peroxide into fly food and in sucrose, respectively. The lifespan was significantly longer for flies fed with paraquat in the fly food than those fed with paraquat in sucrose ^33^. This suggests that nutrients in the fly food are beneficial to flies in their resistance to paraquat toxicity, a finding consistent with a recent report ^33^. Our results show that paraquat did not show any dramatic synergistic effects with mutant hSOD1 whether the mutant protein is expressed in MNs, glia, or in both. Further, expression of an enzymatically inactive form of mutant SOD1 (G85R) ^2^ did not enhance the toxic effect of paraquat. Finally, expression of the *Drosophila* wild-type SOD1 (dSOD1) in MNs, glia, or in both cells does not afford any resistance to paraquat. These results suggest that SOD1 may not be the primary enzyme in flies to protect tissues from attacks by radical oxygen ( ^-^O ^-^) produced by paraquat. It is possible that the systemic production of radical oxygen ( ^-^O ^-^) by paraquat outweighs the dismutase activity of the SOD1 proteins expressed in MN and glial cells. Interestingly, dSOD1 briefly extends the lifespan of D42 and M1B but not of D42+M1B flies fed on H _2_O _2_. We do not know why there is a small difference between paraquat and H _2_O _2_ but speculate that the level of radical oxygen species may be significantly lower in H _2_O _2_ or that H _2_O _2_ causes lethality through mechanisms different from those by paraquat.

Another important finding of this study is the first demonstration of unique interactions between mutant hSOD1 and the environmental factor BMAA in a cell type-specific manner. In motoneurons mutant SOD1 appears to have protective roles in prolonging the lifespan of BMAA-treated flies whereas the same mutant SOD1 enhances the sensitivity to BMAA when expressed in glia. Notably, the protective effect on longevity is also observed for the enzymatically inactive G85R. These results suggest that the neuroprotective role of SOD1 against BMAA is unrelated to the dismutase activity of SOD1. Further, G85R expressed in glia does not further enhance the toxic effect of BMAA, arguing against the idea that BMAA shortens the fly lifespan via an oxidative stress mechanism.

How mutant SOD1 offers neuroprotection in motoneurons but enhances BMAA toxicity in glia remains unclear. It has been proposed that BMAA exerts toxic effects in murine cortical cell lines through activation of NMDA and mGluR5 receptors ^21^. Bruijn and colleagues observed inclusions in astrocytes in a G85R transgenic mouse model, implicating astrocytes as primary targets for mutant-SOD1 mediated damage ^46^. Trotti and colleagues suggest that the failure of astrocyte-mediated glutamate transport may be linked to neurodegeneration ^47^. In an embryonic stem cell-based system motoneurons carrying either the non-pathological human *sod1* transgene or the ALS-linked *sod1* mutant G93A allele showed neurodegenerative properties when co-cultured with G93A glial cells ^14^. Our data are consistent with the notion that mutant hSOD1 plays a major non-cell autonomous role in glia. We favor a ‘glutamate excitotoxicity’ hypothesis in which mutant SOD1 impairs glial function thereby potentiating the effect of BMAA on neurons.

In conclusion, our results provide a new understanding of SOD1 target cell type. While most research showing glial involvement in ALS was done in mouse model ^9, 11, 48^, we are the first to show such involvement in a *Drosophila* model. The interactions of SOD1 and BMAA and H _2_O _2_ in our model represent a non-cell autonomous type of effect on the motor activity and overall survival of the transgenic flies.
