# Supplementary figures and images for: ALS-linked SOD1 in glial cells enhances ß-N-Methylamino L-Alanine (BMAA)-induced toxicity in Drosophila
Source: F1000Res. 2012 Nov 9;1:47. [Version 1] doi: 10.12688/f1000research.1-47.v1 (PMC3945777; doi:10.12688/f1000research.1-47.v1)

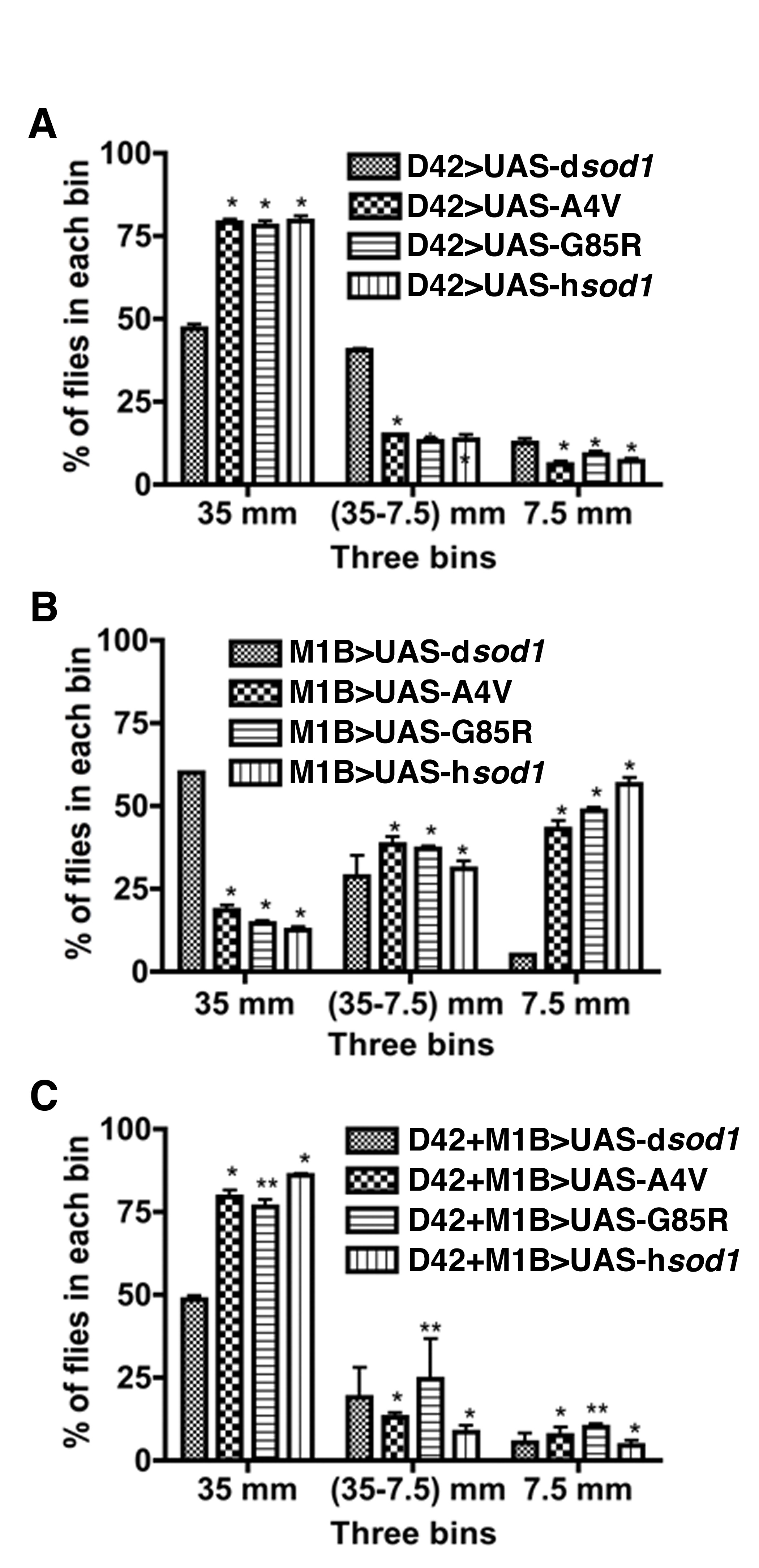

Supplement: Climbing behavior of 60 day-old flies. — Healthy looking 60 day-old male flies were selected for this assay. Ten flies were placed in a 100 ml glass cylinder. Following a brief and gentle tapping, the distribution of flies was then counted in 3 bins at different heights (above or at 35 mm height, between 7.5 mm and 35 mm, and 7.5 mm or lower). Climbing pattern of flies expressing SOD1 in motoneurons (A), glial cells (B), and co-expressing SOD1 in both motoneurons and glial cells (C) are shown. Flies expressing human SOD1 proteins in glial cells show a significant shift of distribution to the bottom of the cylinder compared to M1B>UAS-dsod1 (the control fly) and in comparison with their expression in motoneurons (A) or dual expression in MNs + glia (C). These observations suggest a significant impairment of climbing activities at 60 days in flies expressing human SOD1s in glia. The statistical significances were calculated using Prizm software, Two-way ANOVA and * p [file f1000research-1-222-s0009.tgz › Figure_1_Zhang.jpg]

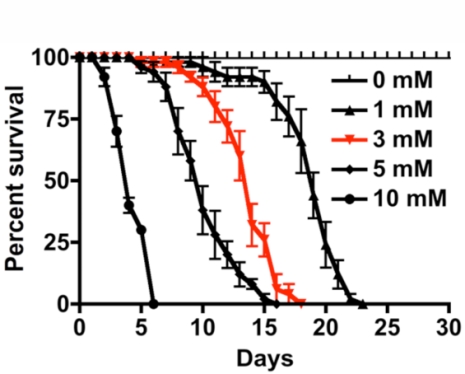

Supplement: Survival curve of wild type (Canton S) Drosophila under various dosages of BMAA. — Five day-old male flies were reared in 1, 3, 5, and 10 mM BMAA diluted in 3% sucrose for up to 30 days (10 flies per vial). The 50% survival time for 1, 3, 5, and 10 mM BMAA was 19, 14, 10, and 4 days, respectively. The sham (0 mM BMAA) treated flies did not show any toxicity during the observation period. The statistics was performed using One-way ANOVA analysis of variance in Prizm software, p [file f1000research-1-222-s0008.tgz › Figure_2_Zhang.jpg]

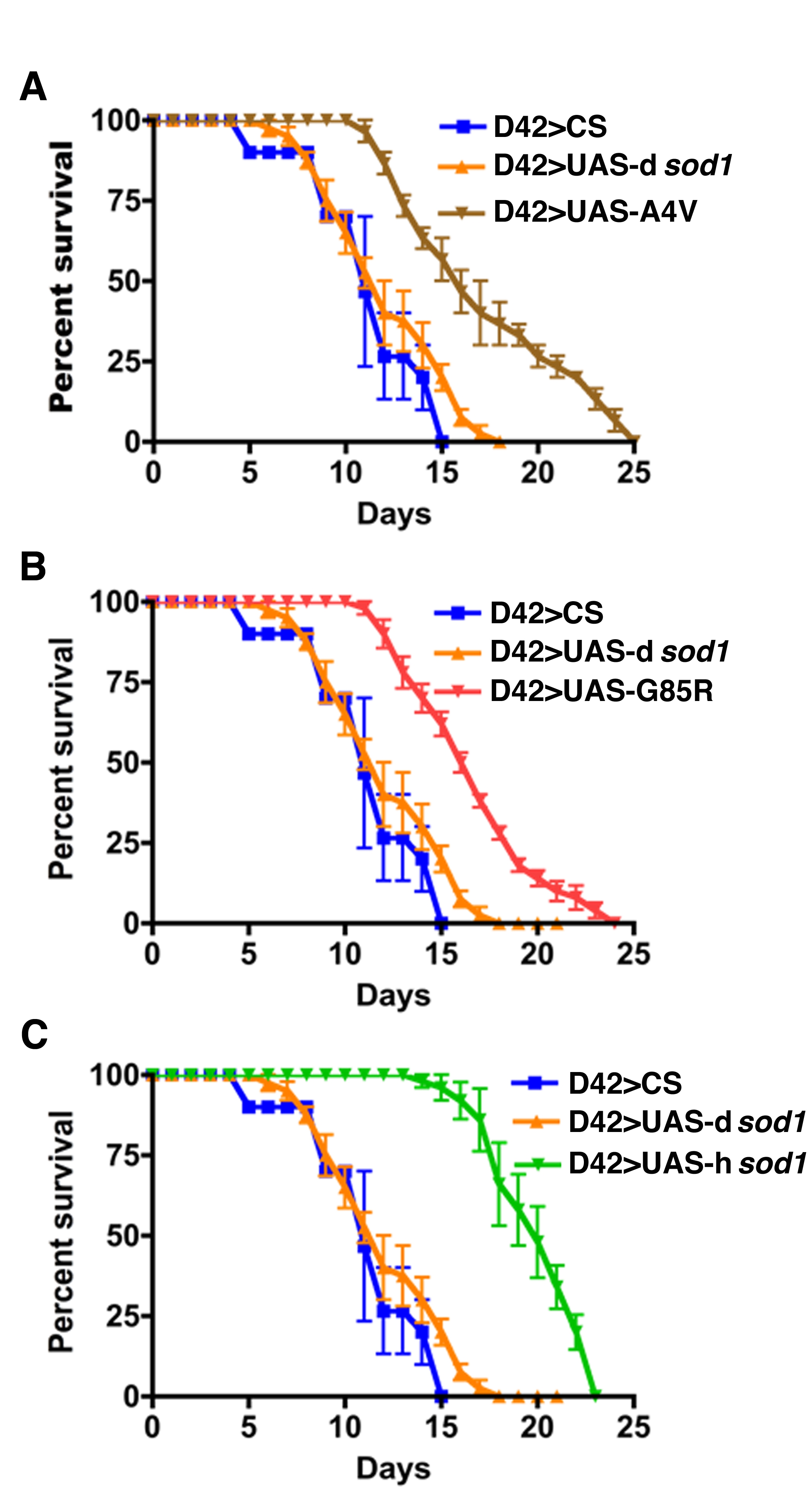

Supplement: Human SOD1 overexpressed in motoneurons confers resistance to BMAA treatment. — Shown are survival rates of 5 day-old male sod1 transgenic flies under 3 mM BMAA treatment (10 flies per vial). Panels A, B, and C represent the survival rate of flies expressing mutant human A4V, G85R, wild type SOD1 proteins and control flies (D42>CS and D42>UAS-dsod1) in motoneurons using the D42-Gal4 driver. Flies expressing the human SOD1s (both the hSOD1WT, and mutants, hSOD1A4V and hSOD1G85R) survived longer compared to the control flies. At the 50% survival rate, the longevity is increased by 33%, 41% and 66 % for A4V, G85R, wt SOD1, respectively, when compared to D42>dsod1 and D42>CS flies. Differences were considered statistically significant if pCS and D42>A4V, respectively; n=50 for the rest of genotypes. [file f1000research-1-222-s0007.tgz › Figure_3_Zhang.jpg]

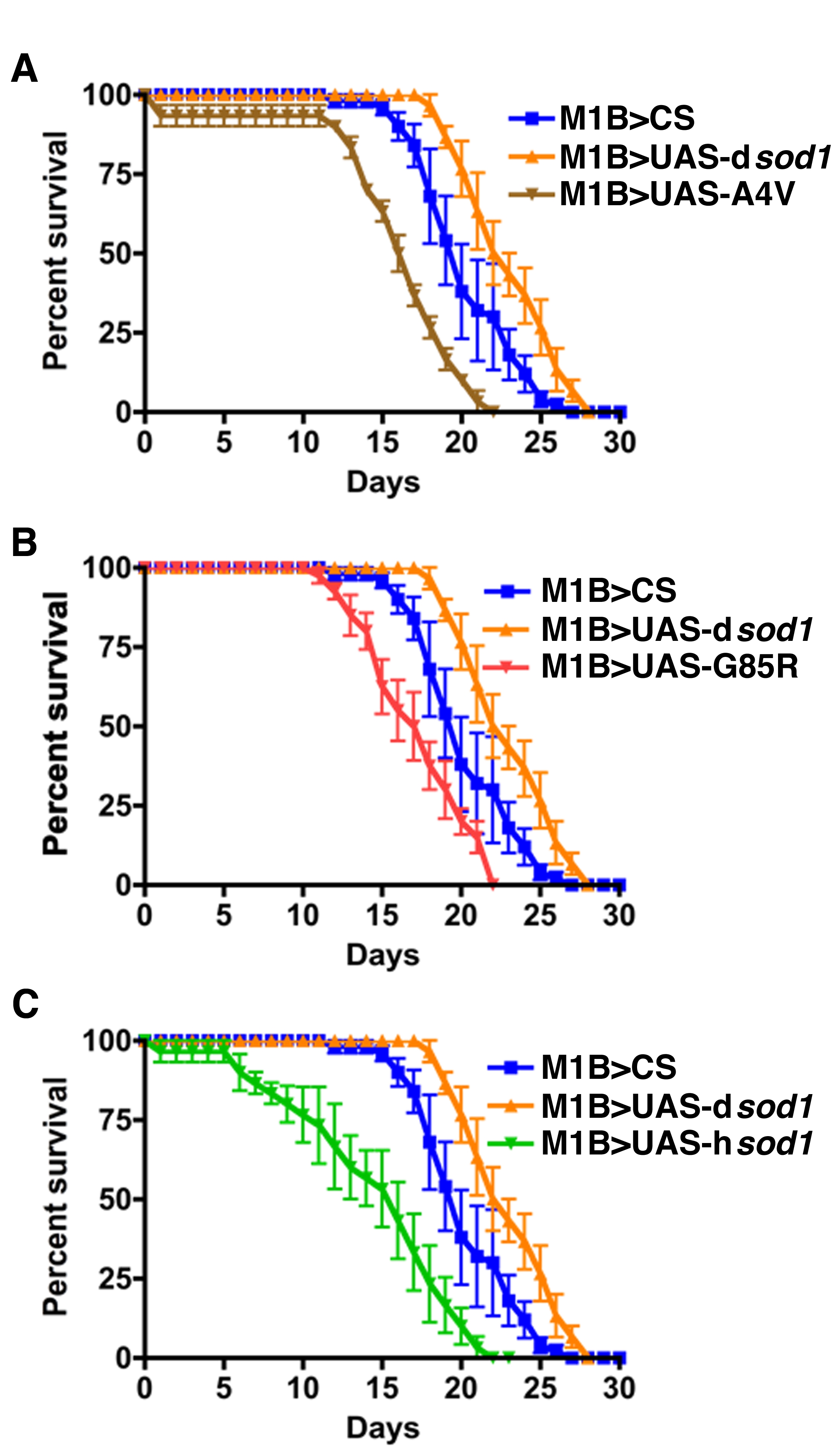

Supplement: Human SOD1 overexpressed in glial cells accelerates fly death by BMAA. — Shown are survival rates of 35 day-old male sod1 transgenic flies under 3 mM BMAA treatment. Panels A, B, and C represent flies expressing mutant human A4V, G85R, and wild type SOD1 proteins, respectively. Like 5 day-old time points (Figure 3, A-C), the SOD1 flies survived longer compared to the controls (CS and dSOD1). The 50% survival rate is increased by 10, 30 and 30% for A4V, G85R, wt SOD1, respectively, when compared to dSOD1 flies. Differences were considered statistically significant if p [file f1000research-1-222-s0006.tgz › Figure_4_Zhang.jpg]

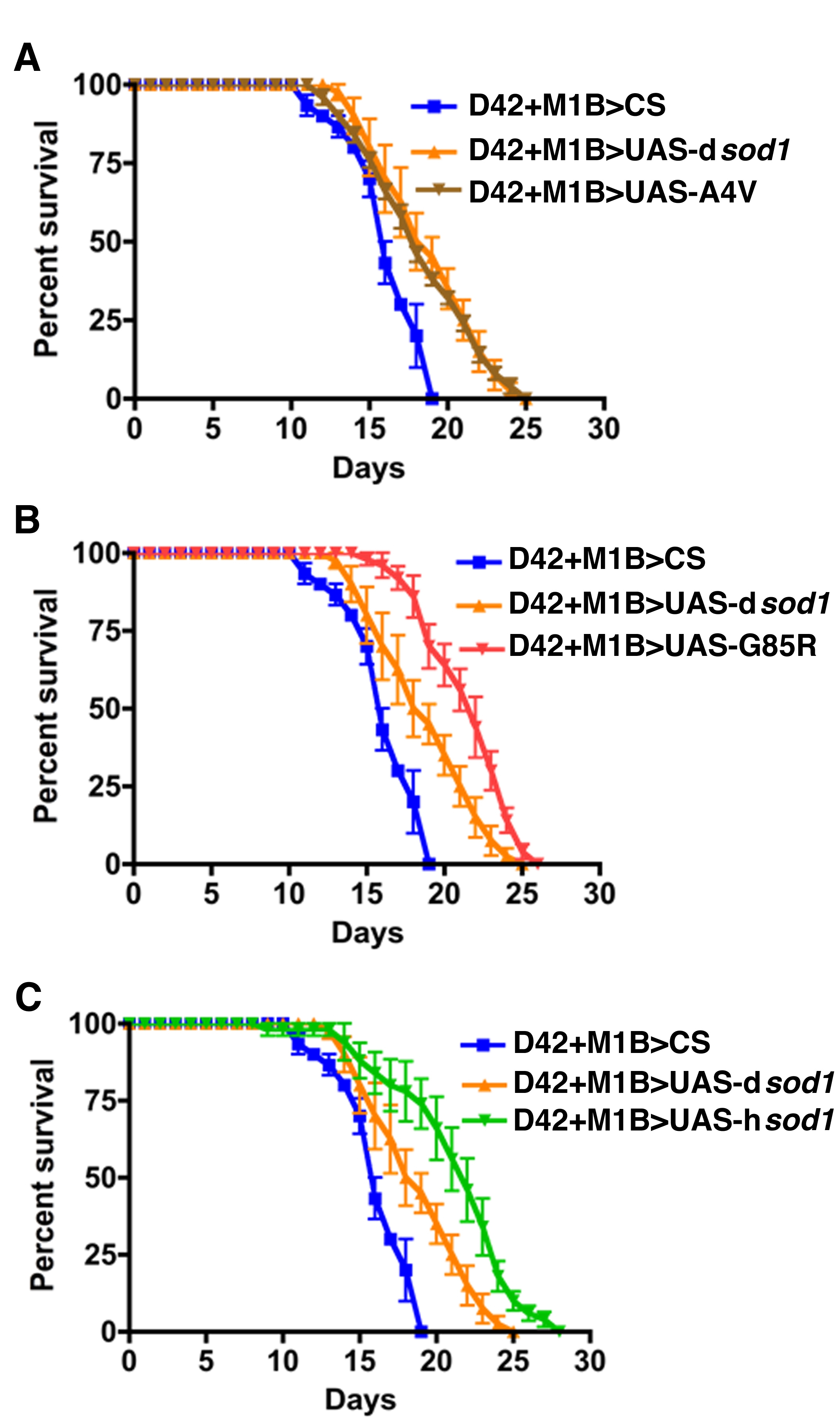

Supplement: Human SOD1 co-overexpressed in motoneurons and glial cells and treated with 3 mM BMAA. — Shown are survival rates of 35 day-old male sod1 transgenic flies under 3 mM BMAA treatment. Panels A, B, and C represent flies expressing mutant human A4V, G85R, and wild type SOD1 proteins, respectively. Like 5 day-old time points (Figure 3, A-C), the SOD1 flies survived longer compared to the controls (CS and dSOD1). The 50% survival rate is increased by 10, 30 and 30% for A4V, G85R, wt SOD1, respectively, when compared to dSOD1 flies. Differences were considered statistically significant if p [file f1000research-1-222-s0005.tgz › Figure_5_Zhang.jpg]

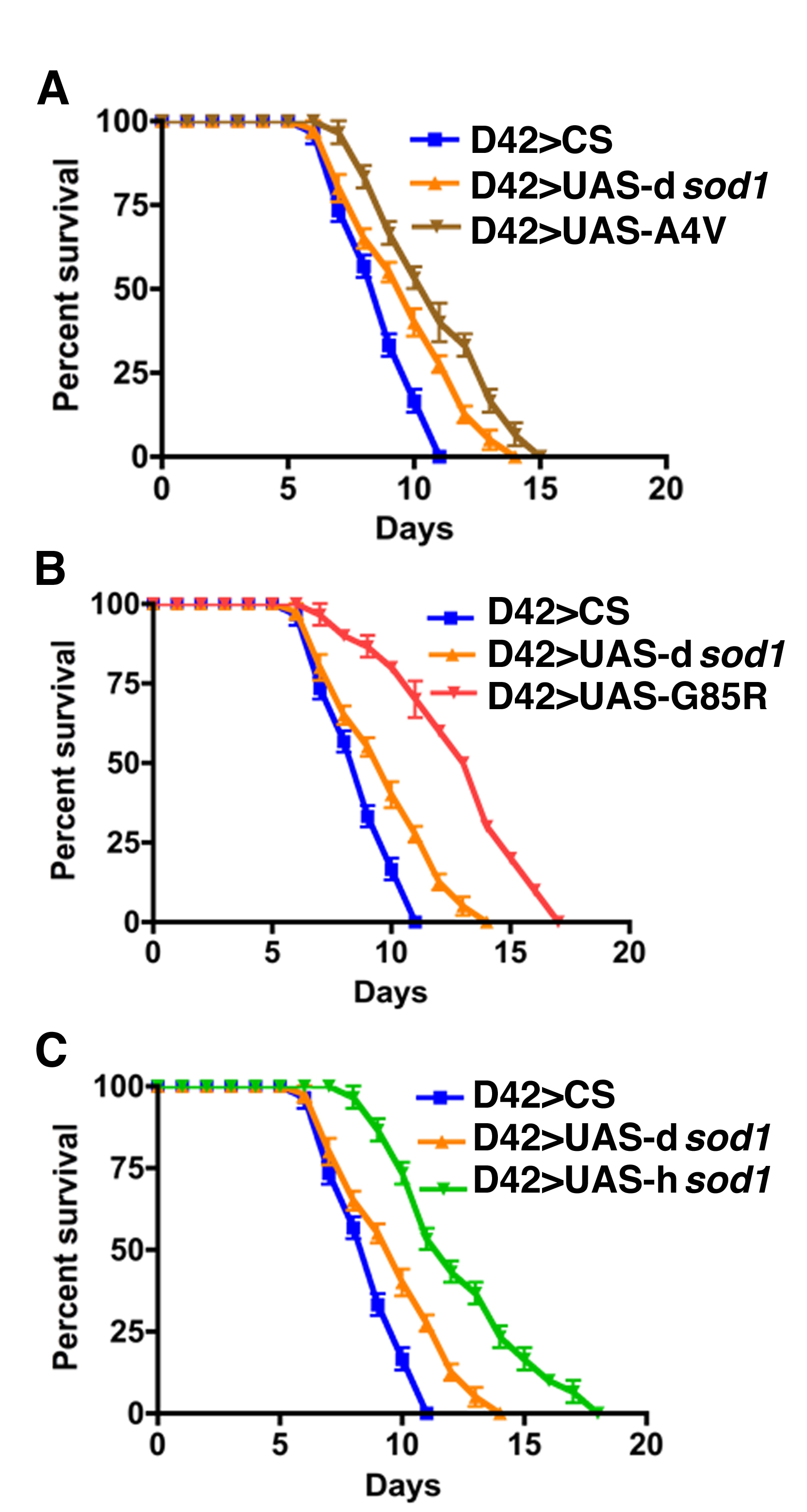

Supplement: Human SOD1 over-expressed in motor neurons confers resistance to BMAA treatment. — Shown are survival rates of 35 day-old male sod1 transgenic flies under 3 mM BMAA treatment (10 flies per vial). Panels A, B, and C represent flies expressing mutant human A4V, G85R, and wild type SOD1 proteins, respectively. Like 5 day-old time points (Figure 3, A-C), the SOD1 flies survived longer compared to the controls (CS and dSOD1). The 50% survival rate is increased by 10, 30 and 30% for A4V, G85R, wt SOD1, respectively, when compared to dSOD1 flies. Differences were considered statistically significant if p [file f1000research-1-222-s0003.tgz › Supplementary_Fig_1.jpg]

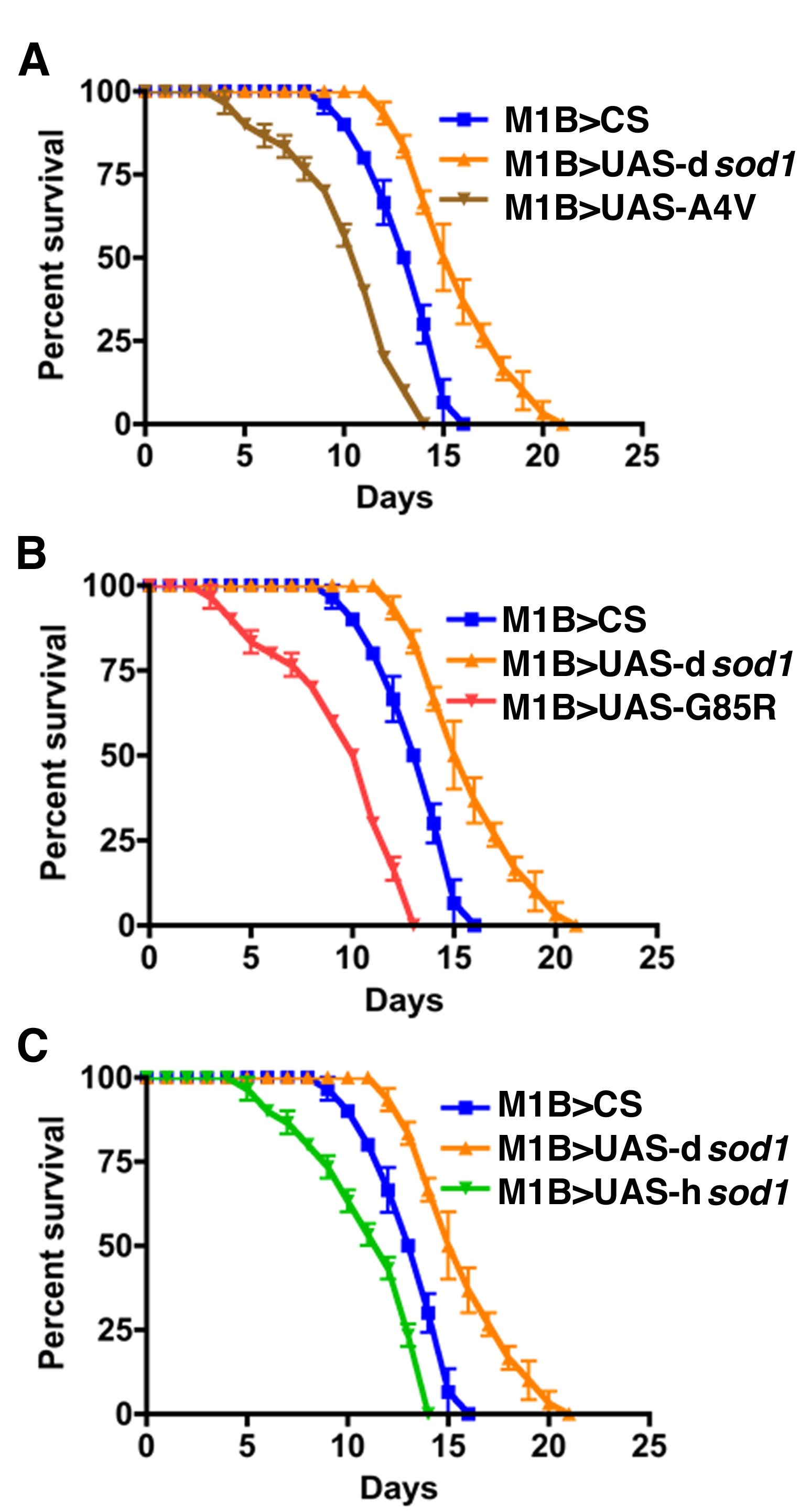

Supplement: Human SOD1 over-expressed in glial cells enhances the effect of BMAA on reducing the longevity of flies. — Shown are survival rates of 35 day-old male sod1 transgenic flies under 3 mM BMAA treatment (10 flies per vial). Panels A, B, and C represent flies expressing mutant human A4V, G85R, wild type SOD1 proteins. The 50% survival rate of A4V, G85R, and hSOD1 when compared with dSOD1 is decreased by 31, 38, and 38%, respectively. Differences were considered statistically significant if p [file f1000research-1-222-s0002.tgz › Supplementary_Fig_2.jpg]

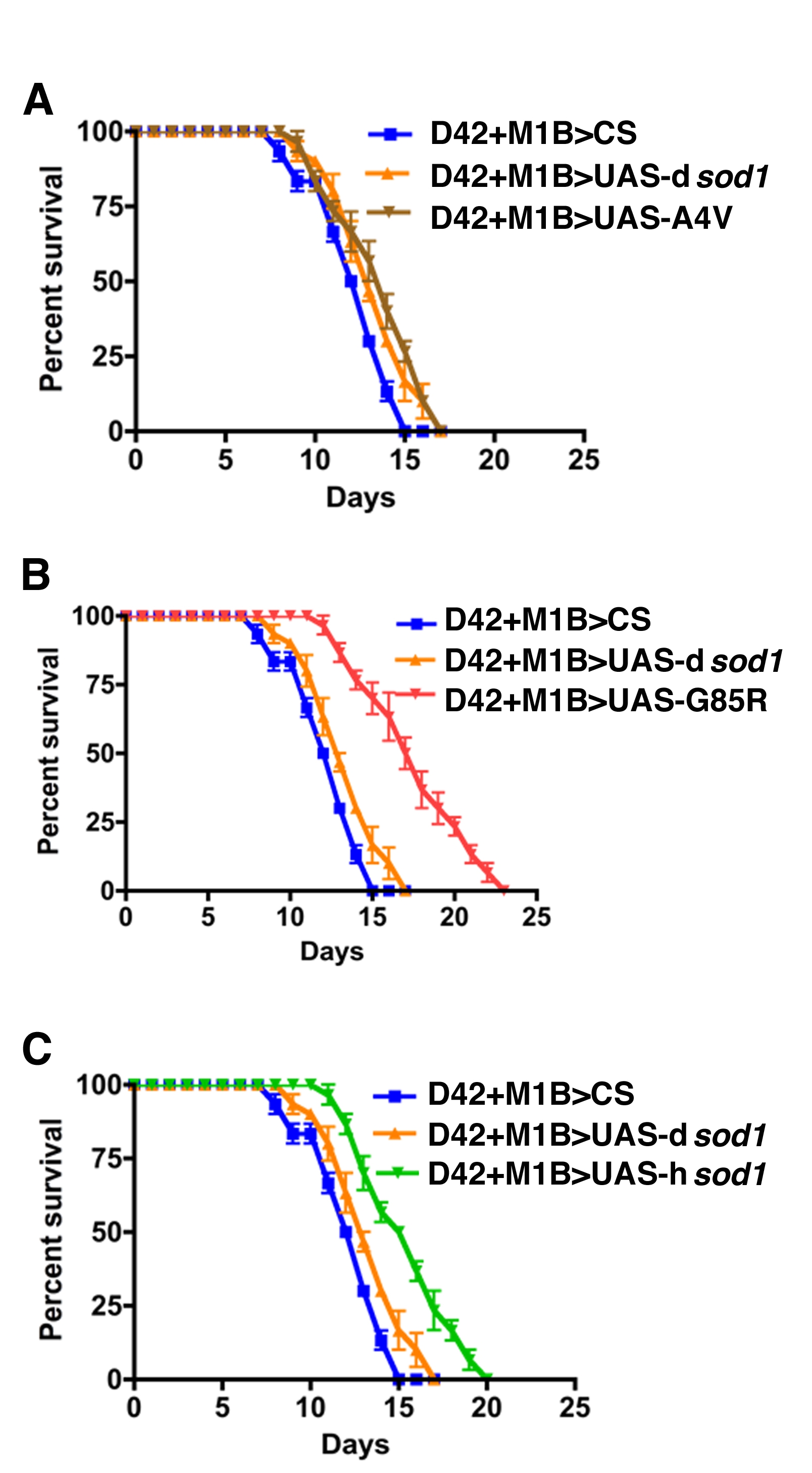

Supplement: Human SOD1 co-overexpressed in motor neurons and glial cells and treated with 3 mM BMAA. — Shown are survival rates of 35 day-old male sod1 transgenic flies under 3 mM BMAA treatment (10 flies per vial). Panels A, B and C represent flies expressing mutant human A4V, G85R, wild type SOD1 proteins. The 50% survival rate of A4V, G85R, and hSOD1 when compared with dSOD1 is increased by 8, 30, and 15%, respectively. Differences were considered statistically significant if p [file f1000research-1-222-s0001.tgz › Supplementary_Fig_3.jpg]

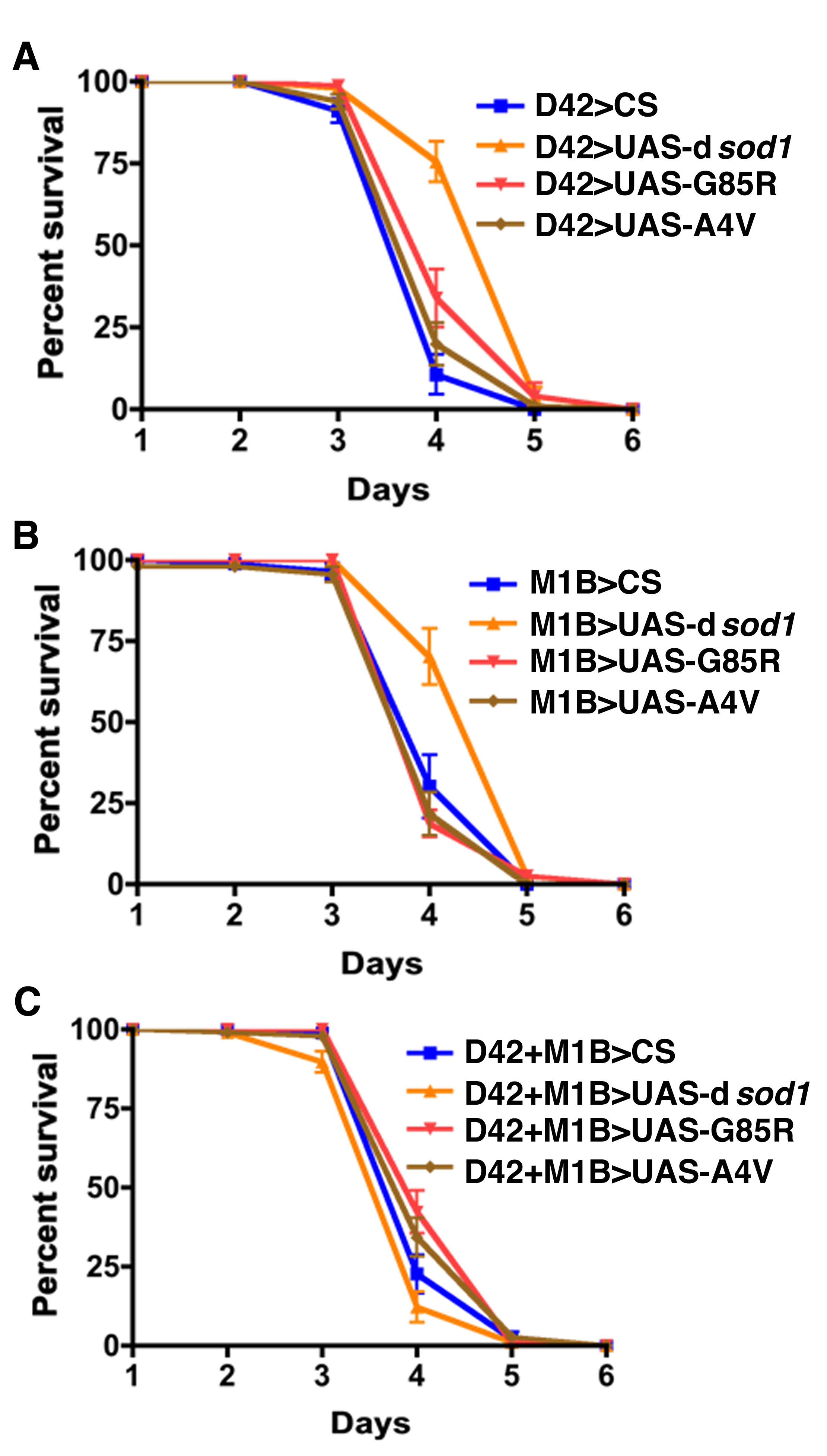

Supplement: Effects of H2O2 on SOD1 transgenic flies. — Five day-old male flies expressing SOD1 proteins in motor neurons (A), glial cells (B) and in motoneurons together with glial cells (C) were treated with 1% H2O2 diluted in 3% sucrose. The results indicate an enhancing effect of H2O2 on mutant SOD1 toxiciy in MNs or glia when compared to driver>UAS-dsod1, but not to driver>CS control. Statistical analysis was performed using a two-tailed ANOVA method in Prizm software and was considered significant if p [file f1000research-1-222-s0004.tgz › Figure_6_Zhang.jpg]

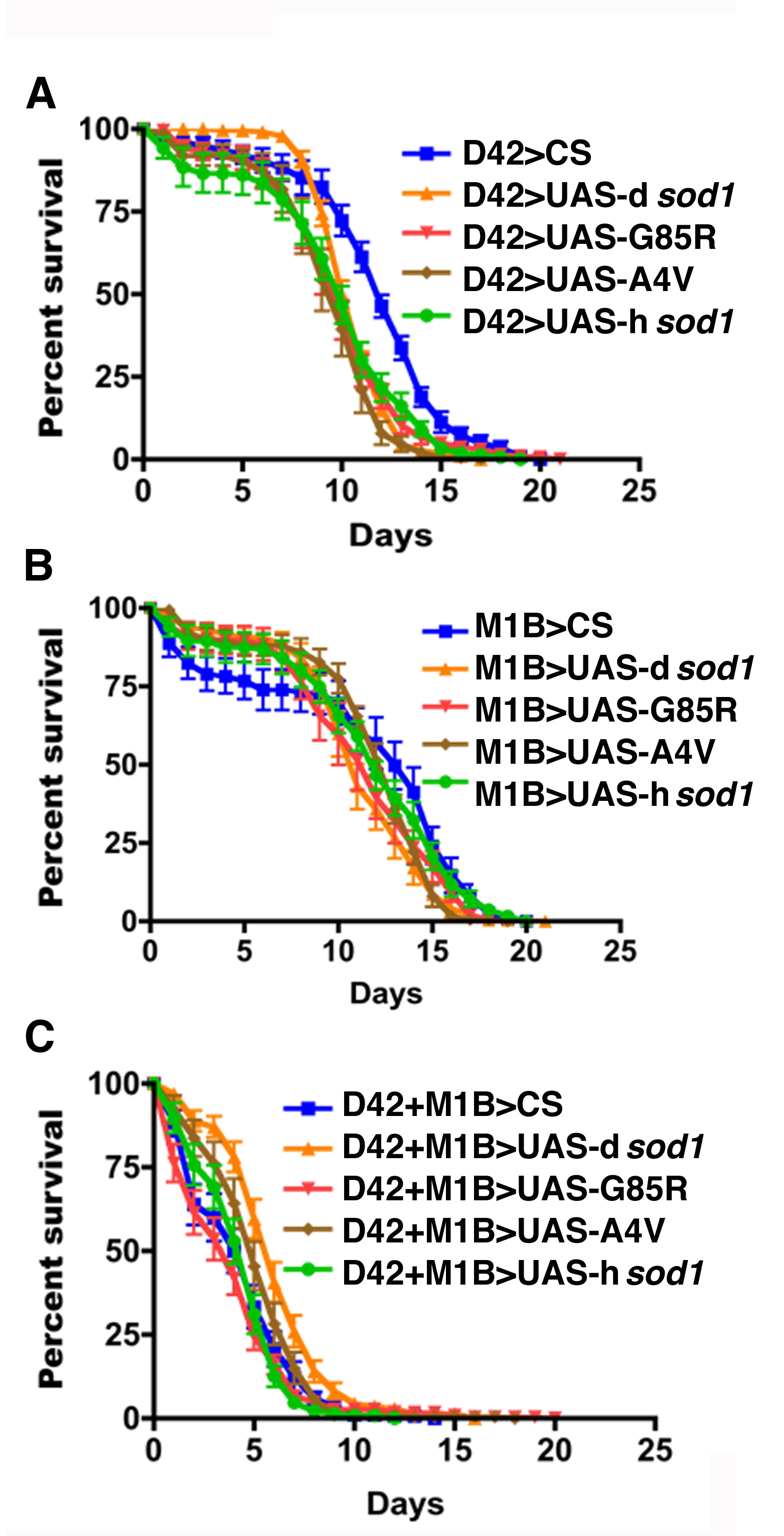

Supplement: Effects of paraquat on SOD1 transgenic flies. — Five day-old male flies expressing SOD1 proteins in motor neurons (A), glial cells (B) and in motoneurons together with glial cells (C) were treated with 20 mM paraquat in fly food (10 flies per vial). The results do not indicate any significant effect of paraquat in SOD1 flies of all genotypes. Total flies uses for D42Gal4>CS, 183; D42Gal4>UAS-dsod1, 194; D42Gal4>UAS-hSOD1A4V, 138; D42Gal4>UAS-hSOD1G85R, 206; D42Gal4>UAS-hSOD1, 231. M1BGal4>CS, 148; M1BGal4>UAS-dsod1, 171; M1BGal4>UAS-hSOD1A4V, 164; M1BGal4>UAS-hSOD1G85R, 144; M1BGal4>UAS-hSOD1, 200. D42+M1BGal4>CS, 148; D42+M1BGal4>UAS-dsod1, 156; D42+M1BGal4>UAS-hSOD1A4V, 177; D42+M1BGal4>UAS-hSOD1G85R, 286; D42+M1BGal4>UAS-hSOD1, 213. [file f1000research-1-222-s0000.tgz › Supplementary_Fig_4.jpg]
