# Supplementary material for: ALS-linked SOD1 in glial cells enhances ß-N-Methylamino L-Alanine (BMAA)-induced toxicity in Drosophila
Source: F1000Res. 2012 Nov 9;1:47. [Version 1] doi: 10.12688/f1000research.1-47.v1 (PMC3945777; doi:10.12688/f1000research.1-47.v1)
Supplement: Peer review history

# Peer review: Referee response for version 1

**Published:** 2012-11-26

**Authors:** Grace Zhai

**Affiliations:** 1 Department of Molecular and Cellular Pharmacology, Miller School of Medicine, University of Miami, Miami, FL, USA

This is an interesting article. One thing to note is that the three panels (A, B, and C) in Figures 3, 4, 5, S1, S2, and S3 should be combined, as it is redundant to show the same data three times in these figures.

I have read this submission. I believe that I have an appropriate level of expertise to confirm that it is of an acceptable scientific standard.

# Peer review: Referee response for version 1

**Published:** 2012-11-15

**Authors:** Joy Goto

**Affiliations:** 1 Department of Chemistry, California State University, Fresno, CA, USA

I have read this submission. I believe that I have an appropriate level of expertise to confirm that it is of an acceptable scientific standard.
